# Gold Nanoparticles in Nanomedicine: Unique Properties and Therapeutic Potential

**DOI:** 10.3390/nano14221854

**Published:** 2024-11-20

**Authors:** Furkan Eker, Emir Akdaşçi, Hatice Duman, Mikhael Bechelany, Sercan Karav

**Affiliations:** 1 Department of Molecular Biology and Genetics, Çanakkale Onsekiz Mart University, Çanakkale 17100, Turkey; furkan.eker@stu.comu.edu.tr (F.E.); emirakdasci@gmail.com (E.A.); hatice.duman@comu.edu.tr (H.D.); 2 Institut Européen des Membranes (IEM), UMR 5635, University Montpellier, ENSCM, CNRS, F-34095 Montpellier, France; 3 Functional Materials Group, Gulf University for Science and Technology (GUST), Masjid Al Aqsa Street, Mubarak Al-Abdullah 32093, Kuwait

**Keywords:** gold nanoparticle, drug delivery, bioimaging, biosensor, antibacterial, photothermal therapy, photodynamic therapy, anticancer, LSPR, SERS

## Abstract

Gold nanoparticles (NPs) have demonstrated significance in several important fields, including drug delivery and anticancer research, due to their unique properties. Gold NPs possess significant optical characteristics that enhance their application in biosensor development for diagnosis, in photothermal and photodynamic therapies for anticancer treatment, and in targeted drug delivery and bioimaging. The broad surface modification possibilities of gold NPs have been utilized in the delivery of various molecules, including nucleic acids, drugs, and proteins. Moreover, gold NPs possess strong localized surface plasmon resonance (LSPR) properties, facilitating their use in surface-enhanced Raman scattering for precise and efficient biomolecule detection. These optical properties are extensively utilized in anticancer research. Both photothermal and photodynamic therapies show significant results in anticancer treatments using gold NPs. Additionally, the properties of gold NPs demonstrate potential in other biological areas, particularly in antimicrobial activity. In addition to delivering antigens, peptides, and antibiotics to enhance antimicrobial activity, gold NPs can penetrate cell membranes and induce apoptosis through various intracellular mechanisms. Among other types of metal NPs, gold NPs show more tolerable toxicity capacity, supporting their application in wide-ranging areas. Gold NPs hold a special position in nanomaterial research, offering limited toxicity and unique properties. This review aims to address recently highlighted applications and the current status of gold NP research and to discuss their future in nanomedicine.

## 1. Introduction

Nanomaterials have become one of the most significant materials utilized in wide-ranging applications. Nanoparticles, in this case, have proven their significance in nanotechnology with unique characteristic features. They possess significant reactivity through their large surface area, tunable size, modifiable surface for wide-ranging applications, and shape that can alter physicochemical properties [1].

**Figure 1:**
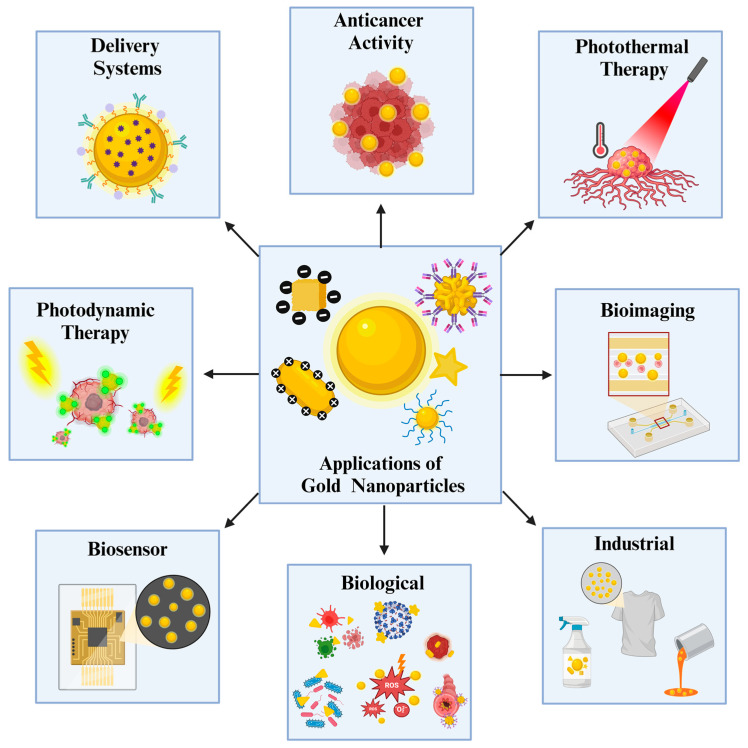
Applications of gold NPs in various fields [3,5].

Gold NPs possess unique optical properties that are easily modified with alterations of physicochemical properties [2]. They are found in various shapes, such as nanocubes, nanorods, triangles, nanostars, and nanospheres, and different sizes that affect their color, which is utilized in many applications [3]. In addition, their surface chemistry can be easily altered and conjugated with various molecules, which expands their application to many areas, such as drug delivery and photothermal therapies (PTT) (Figure 1) [4].

Gold NPs’ unique physicochemical properties, including strong LSPR, ease of functionalization with a wide range of biomolecules, antimicrobial characteristics, and anticancer and antioxidant activity, have been frequently exploited in the current literature. In addition, attributes that originate from their nature, such as biocompatibility, inertness, and low toxicity, create an advantageous state for future uses where gold NPs are employed. These unique properties are highly expressed in wide-ranging applications, especially in imaging, sensor development, and cancer radiotherapy as an efficient radiosensitizer (Figure 1) [6]. Thanks to their unique physicochemical properties and significant X-ray absorption, they have been used as a radiosensitizer in many *in vitro* and *in vivo* studies [7].

**Figure 2:**
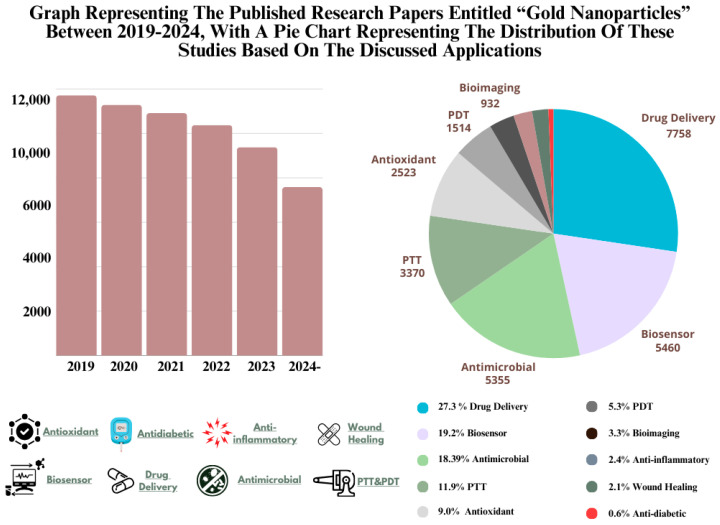
Graph representing the published research papers that include “gold nanoparticles” in their title for the last 5 years, with a pie chart showing the distribution of applications based on the discussed sections [8].

When the total number of published articles in the last five years is observed, there is notable consistency in research numbers during the first four years (Figure 2). Even though there is a slight decline in the last two years, it is evident that the field remains active with approximately 10,000 publications annually. This can be explained by the number of published patents and the current challenges, which are evaluated in later sections.

According to the presented pie chart, drug delivery and biosensor application of gold NPs lead the majority of research compared to the other applications that are discussed in this review. Moreover, antimicrobial activity, comprising antibacterial, antifungal, and antiviral studies, manages to closely follow these areas. This distribution is heavily influenced by the unique optical properties and efficient carrier characteristics of gold NPs.

Due to their unique optical characteristics, the distribution of their application shows a great difference compared to other types of metals NPs. For instance, silver NPs, one of the most studied types of metal NPs in the literature, are known to have significant antimicrobial activity [9]. Based on this, while silver NPs are predominantly utilized in antibacterial and wound healing studies [10], gold NPs seem to be tested in delivery, imaging, and anticancer studies, including photodynamic therapy (PDT) and PTT, as the distribution is clearly visible in the recent clinical trials [11]. That is the reason why review articles focusing on recent applications of and advances in gold NPs are very important. The distribution analysis of the currently published research works, pointing out the challenges, and discussion of future perspectives are important to guarantee further development in gold NP applications, considering the highly active status of this field. In particular, we have addressed the currently growing application areas and the ones that need attention, suggesting research gaps that need addressing. These are highly critical in allowing wider clinical and industrial applications. This review provides a comprehensive overview of well-explored areas, especially drug delivery and biosensors, and overlooked applications like bioimaging and anti-inflammatory uses, therefore offering valuable insights into potential research directions that could drive innovation in the future of NP research.

## 2. Properties of Gold Nanoparticles

**Figure 3:**
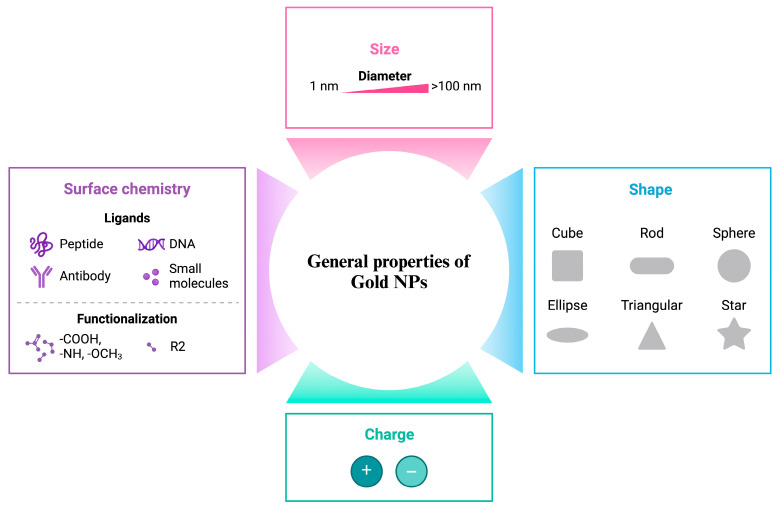
General properties of gold nanoparticles [13].

In nanomedicine-based applications, the physicochemical properties of NPs, such as surface modification, size and shape determination, and surface charge density, play a major role in the determination of their activities [12]. Gold NPs exhibit diverse properties that significantly alter their biological activity in these areas (Figure 3).

Size is a fundamental characteristic influencing the properties of all types of NPs. For gold NPs, size influences several critical factors essential to their applications. The size of gold NPs affects their efficiency in initiating cellular entry. For instance, small gold NPs (up to 15 nm) are readily absorbed by intestinal epithelial cells, whereas larger gold NPs (up to 100 nm) can still penetrate epithelial cells but with reduced efficiency [14]. The optical properties of gold NPs can vary significantly depending on their size. For instance, a comparative study revealed that 70 nm spherical gold NPs demonstrated the most efficient absorption compared to their counterparts ranging from 20 to 100 nm [15].

The shape of the particles is another major determinant of the activity of NPs. Gold NPs can be synthesized in various shapes, such as spherical, rod-shaped, and nanowires, primarily depending on the pH and temperature during synthesis [5]. Similar to other physicochemical properties, the mechanism and efficiency of gold NPs’ cellular uptake are heavily influenced by their shape. For instance, an *in vitro* study demonstrated that gold nanotriangles exhibited the highest cellular uptake, followed by gold nanorods, compared to nanostars [16]. Additionally, the biodistribution of gold NPs can vary significantly depending on their shape. While both spherical and nanostar-shaped gold NPs accumulate in liver tissue (in different regions), only nanostar-shaped gold NPs are found in high concentrations in the lungs [17]. These factors are crucial not only for site-specific applications, such as anticancer therapy and drug delivery, but also for evaluating their toxicity potential.

Surface charge is also a critical factor influencing NPs’ cellular uptake and interaction, which are particularly important in most gold NP applications. Therefore, their surface chemistry can be modified to achieve optimal surface charge density, enhancing cellular interaction when necessary [18]. Depending on their charge, gold NPs can exhibit diverse biodistribution, emphasizing the importance of surface charge density in relation to potential toxicity and application efficiency. For example, positively charged gold NPs tend to accumulate in liver hepatocytes, whereas negatively charged particles exhibit broader tissue distribution [19]. Furthermore, the interaction of gold NPs with the immune system is significantly influenced by their charge density [20].

Another key surface property of gold NPs is their ability to be modified with a diverse range of agents. Surface modification of gold NPs is crucial not only for applications like PTT and PDT-based treatments, drug delivery systems, and anticancer research but also for enhancing their stability to achieve the desired therapeutic outcomes. Regarding the importance of the surface chemistry of NPs, a brief discussion on this factor is essential. Physicochemical properties of NPs are influenced by the capping agents used during the synthesis [21]. Various capping agents, such as chitosan, bovine serum albumin, and polyethylene glycol, have been employed in the nanomedicine applications of NPs [21]. These agents define the surface chemistry of gold NPs by determining specific characteristics. Redox potential is a crucial characteristic that is significantly influenced by the choice of capping agents [22,23]. It is a key factor for gold NPs, given its impact on biomedical applications.

LSPR is one of the most prominent characteristics of gold NPs, significantly tailoring their preferred applications. These particles can initiate unique interaction with their free electrons under light irradiation. Due to their selective physicochemical properties, the LSPR effect enables a surface-enhanced spectrum, facilitating the efficient application of gold NPs in PDT and PTT treatments [24]. The efficiency of these spectral signals can vary depending on the shape of the gold NPs, influencing the preferred shape in specific applications as discussed in the subsequent sections. Shapes that exhibit highly localized regions, such as nanotriangles and nanostars, can provide higher local field enhancement, whereas less structured shapes like spherical particles offer lower enhancement [25].

## 3. Applications of Gold Nanoparticles

Thanks to the unique and significant characteristics of gold NPs, they have been widely used in nanomedicine with a great impact. With tunable surface chemistry, significant physicochemical-dependent optical properties, especially LSPR, and higher tolerability with good biocompatibility, gold NPs are utilized in a wide range of biomedical applications. As shown in Figure 2, gold NPs are predominantly applied in drug delivery, biosensors, and PDT/PTT-based therapies, primarily focusing on anticancer research. In this section, we highlight and provided detailed examples of efficient localized heat generation upon light irradiation, the potential of surface modifications in antimicrobial treatment and drug delivery, the unique LSPR property for sensor development, and areas requiring further research, such as wound healing and bioimaging applications.

### 3.1. Delivery Systems

**Figure 4:**
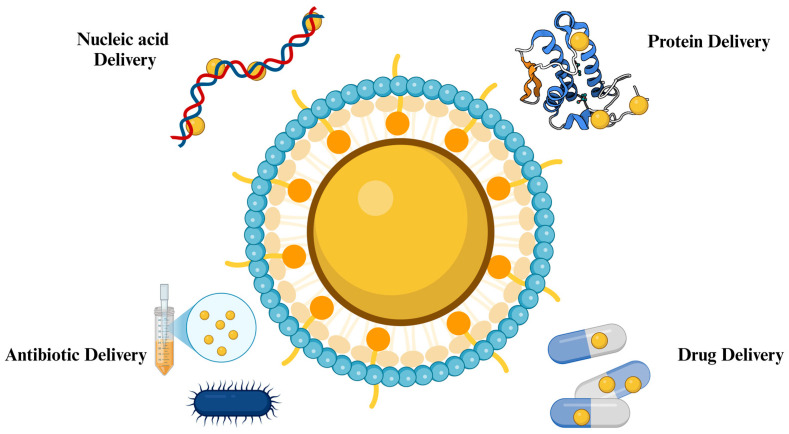
Drug delivery application of gold NPs [29].

Gold NPs are capable of delivering different types of biomolecules, including recombinant proteins, wide-ranging drugs, and nucleotides [26]. They possess a high capacity to be functionalized and the possibility to be altered, in both size and shape, which allows their utilization in the delivery of not only small but also large biomolecules (Table 1) [27]. This is why various structures of gold NPs, such as nanorods, spheres, and composites, are utilized in drug delivery applications [28]. In addition, they possess susceptibility to penetrate tumor cells more than the blood vessels of vital tissues, such as the heart and lungs [7]. Therefore, gold NPs are currently used in various delivery systems in several areas (Figure 4).

**Table 1:** Recent drug delivery applications of gold NPs.

Application	Synthesis Method	Properties	Results	Reference
*In vitro* cervical cancer treatment with curcumin conjugation	Chemical synthesis	Average size of 7 nm ± 2.29 nm. Spherical morphology. SPR peaks at 525 nm.	- Enhanced bioavailability of curcumin against HeLa cells. - Insignificant toxicity in the zebrafish embryo model. - Significant radiosensitizing activity. - Enhanced intracellular reactive oxygen species (ROS) generation, apoptotic signals, and DNA damage.	[30]
Co-delivery with miRNA-33a to MCF-7 breast cancer cells	Purchased from Nanosany with >95% purity	Size ranges from 50 to 100 nm. Spherical morphology. (Properties of modified gold NPs.)	- Enhanced gene expression in co-delivery. - Enhanced inhibitory activity against breast cancer cells. - Potential synergistic effect through modulation of signaling pathways. - Increased apoptosis rate.	[31]
Enhanced delivery of bleomycin in electrochemotherapy	Chemical synthesis	13 nm size. Spherical morphology. LSPR peak at 521 nm.	- Enhanced cell permeabilization by 40%. - The electric field of 0.9 kV/cm and 1–100 kHz protocols demonstrated significant cytotoxicity by gold NP involvement.	[32]
Delivery of chlorpromazine	Chemical synthesis	Average size of 15 nm and 55 nm. Quasi-spherical morphology.	- Enhanced activity of chlorpromazine in cytotoxicity assays against human (COLO 679) and murine (B16-F0) melanoma cells. - Inhibition of mitochondrial activity and disruption of the cell membrane.	[33]
Delivery of colistin	Chemical synthesis	Average size of 44.34 ± 1.02. Absorbance peaks at 300 ± 0.2 and 515 ± 0.3 nm. (Values from chitosan capped particles.)	- Average drug loading efficacy of 76.4%. - A consistent drug-release profile. - A developed metered-dose inhaler efficiently destroys bacteria over a 12 h period.	[34]
Delivery of phosphazene, delivery of yeast RNA	Chemical synthesis	Spherical morphology.	- Efficient, controlled and long-term release profiles for both drug and RNA delivery. - Antibacterial and antifungal activity.	[35]
**Nucleic acid DNA RNA**				
Delivery of anti-Glut1 SiRNA	Chemical synthesis	Approximate size of 14 nm. Uniform morphology. LSPR peak at 520 nm (red-shifted to 528 nm). (Values from SiRNA containing particles.)	- Significant reduction in Glut1 expression. - Promotion of apoptosis through glucose starvation and ROS cascade signaling. - Inhibition of cancer cell proliferation and tumor growth (*in vivo*). - Induction of apoptosis.	[36]
Delivery of *Fluc* mRNA	Chemical synthesis	Size between 11.52 nm and 12.97 nm. Spherical morphology. Absorption peak at 520 nm	- Significant expression of the luciferase gene compared to naked mRNA delivery.	[37]
SiRNA delivery	Commercially purchased	Size ranging between 20 and 30 nm. Spherical morphology. SPR peak at 520 nm.	- At pH 5.5, green fluorescent protein knockdown levels decreased to 65% in HeLa cells.	[38]
Delivery of *Fluc*-zetagreen reporter genes Delivery of plasmid DNA and synthetic mRNA of SARS-CoV-2 S protein	Chemical synthesis	Mean size of 53 nm. Nanostar morphology. Absorbance peak at 630 nm.	- Enhanced transfection efficiency of *Fluc* gene delivery by increased gold NP-included nanocomposite concentrations in several cell lines. - Significant transfection efficiency of mRNA (at 1000 ng) and DNA (at 250 ng) of S protein by gold NPs.	[39]
**Protein**				
Delivery of SARS-CoV-2 spike protein	Chemical synthesis	Size of 50 nm. Spherical morphology. SPR peak at 529 nm.	- Induced IgG levels. - Induced neutralizing antibody levels. - Significant levels of IFN-γ. (All levels observed in mice.)	[40]
Delivery of atrial natriuretic peptide	Chemical synthesis	Size of 22.34 ± 0.54.	- Significant reduction of tumor formation capacity of retinoblastoma cells from >75% to <50% and <25%. - Significant reduction of tumor formation capacity in mice when the particles are topically administered.	[41]
Antimicrobial peptide delivery	Chemical synthesis	Size of 10 nm. SPR band at 518.5 nm.	- Significant reduction in MIC and MBC values, an average of 200-fold. - 20-fold faster killing capability in bacterial killing kinetics. - 17-fold lower minimum biofilm eradication concentration (MBEC).	[42]
**Antibiotic**				
Delivery of ciprofloxacin	Chemical synthesis	Approximate size of 13 nm. Spherical morphology. Absorption peak at 520 nm.	- Significant reduction in MIC values when the ciprofloxacin administered with gold NPs (2 to 4 times reduction). - Increased zone of inhibition by approximately 4 mm. - Significant biofilm inhibition (from 36.30 nm to 12 nm in analysis on nebulizer masks) and antioxidant capacity (84.66%).	[43]
Conjugation of amikacin for contact lens preservation	Chemical synthesis	Average size of 21 nm. Spherical morphology. Absorption peak at 520 nm.	- Significant reduction in MIC with conjugation on NPs (2 to 4 times reduction). - More than 50% increase in inhibitory zones. - Efficient prevention of biofilm formation in contact lenses (approximate 2–4 times reduction). - Antioxidant capacity by 87.40 at the highest concentration, 100 µg/mL.	[44]
Enhanced antimicrobial activity of berberine	Chemical synthesis	Average size of 49.38 nm. Spherical morphology. Absorption peak at 520 nm.	- 72% maximum release at 72 h. - Nearly 50% decrease in MIC values. - Enhanced bactericidal activity through ROS-mediated membrane disruption and DNA damage. - Approximately 2-fold higher antibiofilm activity by 73.35%. - Significant enhancement of wound healing percentage in mice, with only 2.7% bacteria survival rate.	[45]

#### 3.1.1. Delivery for Cancer Treatment

The drug delivery capacity of gold NPs is commonly used to deliver anticancer drugs. They are included in different types of anticancer drug delivery systems, including light-responsive, pH-based, and glutathione-responsive with the attachment of different therapeutic molecules in various shapes and sizes [46]. In addition to delivering the drug at the target site with an efficient drug release profile, gold NPs can indirectly enhance the activity of the drug. Since the targeting and internalization of the drug will be enhanced, the anticancer drug’s overall activity will be increased. For example, gum karaya-stabilized gold NPs were used to deliver an anticancer drug, gemcitabine hydrochloride, which was loaded onto the surface of the NPs [47]. The surface coating of the drug exhibited 19.2% efficiency. At three various doses (0.1, 0.5, and 1 μg/mL), A549 human lung cancer cells were treated with the anticancer drug with and without the NPs. The cell viability of the cancer cells decreased by nearly 10% in all concentrations when administered with the gold NPs (46.8% to 35.1% at the highest concentration). A significant reduction in colony formation was also observed in the colony formation inhibition assay. To test the efficiency of the drug, the determination of intracellular ROS levels through fluorescence imaging was performed. The anticancer drug exhibited higher fluorescence intensity when combined with gold NPs compared to its sole administration.

The size and structure of the NPs influence the efficiency of the drug delivery of the gold NPs. It has been discussed that the efficiency and internalization of the gold NPs can be highly affected by the size, surface chemistry, and shape of the NPs [48]. As an example, the shape-dependent cytotoxicity of the gold NPs was demonstrated in a research study [49]. Three types of gold NPs, nanospheres, nanorods, and nanostars, were tested against multiple types of pancreatic and bone cancer cells. The results demonstrated that nanostar-shaped gold NPs exhibited the highest cytotoxicity; meanwhile, nanospheres showed the least potential as an anticancer agent.

A similar dependency is also observed in the size of the NPs. For instance, a pH-sensitive drug delivery system for methotrexate was developed for breast cancer treatment, involving two different structures of gelation-coated gold NPs (spherical 50 and 100 nm; nanorod 20, 50, and 100 nm) [50]. *In vitro* drug release revealed that small-sized gold NPs, independent of the structure, exhibited a higher release rate at 5.4 pH. There was an observable change between the two structures of gold NPs in the cell viability test on MCF-7 cells. The spherical gold NPs demonstrated similar cytotoxicity when compared to the control group. In the drug-loaded form, a significant decrease in the cell viability, higher than in the free drug group, was observed. Additionally, the highest results were obtained in the 50 nm size. Conversely, nanorod-shaped gold NPs were highly toxic to cancer cells compared to spherical-shaped ones; meanwhile, the gelatin coating decreased the cytotoxicity. Similarly, drug-loaded nanorods efficiently destroyed breast cancer cells compared to the free drug group. The overall results concluded that nanorod-shaped gold NPs were more efficient as drug carriers than spherical-shaped ones.

Gold NPs, especially nanorods and nanoshells, are used in PTT-based cancer studies (due to their absorption properties) as anticancer drug carriers [51]. The PTT-based anticancer and antitumor applications of gold NPs are evaluated in detail in the subsequent sections. Still, its drug delivery perspective is briefly given in this section. The PTT with gold NPs can alter the environment and increase the cellular permeability and uptake of both the encapsulated drug and the NP itself, which leads to enhanced cytotoxic activity [52].

Niikura *et al*. demonstrated efficient drug release from gold NP vesicles through light irradiation [53]. Doxorubicin-carrier gold NP vesicles were tested *in vitro* on HeLa cells with and without laser irradiation. The irradiation group induced cell death up to 50%; meanwhile, normal treatment did not manage to alter cell viability. Significant morphological changes in HeLa cells were observed in bright-field images; however, this was not the case for other groups (unloaded and not irradiated). A similar approach, a combination of anticancer drug delivery with near-infrared (NIR) irradiation, was demonstrated by Kadkhoda *et al*. [54]. Paclitaxel-loaded aptamer-conjugated gold NPs were modified with poly (ethylene glycol) (PEG) and demonstrated an efficient NIR and pH-dependent drug release. The efficiency of drug encapsulation was determined to be 86%. To determine the influence of pH and irradiation on the drug release ratio, three different pH levels (5.5, 6.5, 7.4) with and without exposure to irradiation were observed. The highest drug release percentage was observed in the lowest pH levels with irradiation. Additionally, all drug release percentages in each pH level were higher with NIR by approximately 10%. The most efficient power of NIR was found to be 160 mW/cm^2^. The *in vitro* cytotoxicity analysis showed a significant decrease in cell viability with NIR compared to non-irradiated NPs. After 3 h, 96% of the NPs internalized into the MUC-1 positive cells, higher than the MUC-1 negative cells (by nearly 2-fold), indicating the targeted activity of the NPs. The modification of the gold NPs caused a significant increase in the apoptosis ratio from 12.8% to 41.49%. Gene expression levels also demonstrated that aptamer conjugation enhanced the apoptosis induction of gold NPs.

#### 3.1.2. Nucleic Acid Delivery

**Figure 5:**
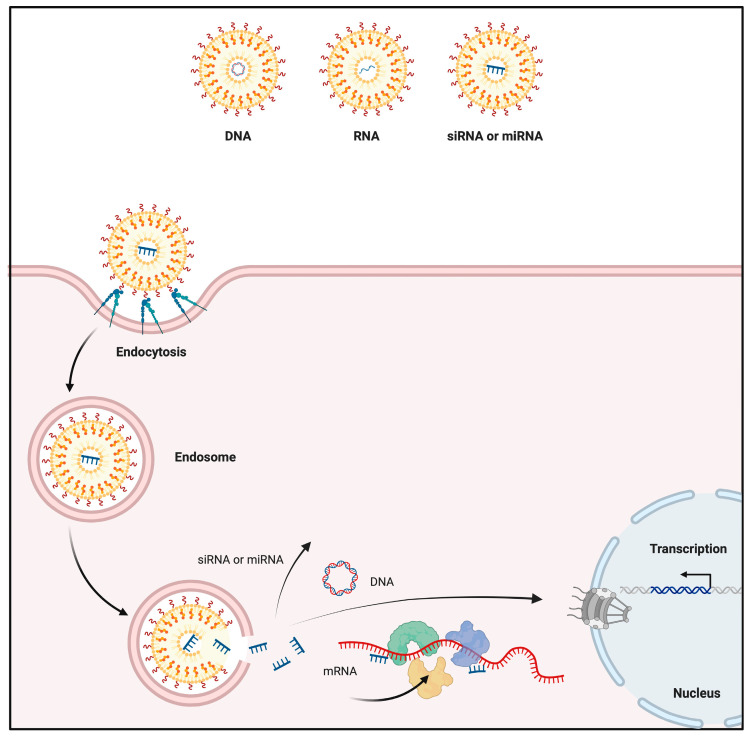
Nucleic acid delivery mechanism of gold NPs. Through endocytosis, functionalized gold NPs effectively transport nucleic acids into cells, and surface alterations improve targeting. The nucleic acids are released into the cytoplasm by endosomal escape mechanisms after internalization, providing opportunities for immunotherapy and gene therapy [61].

Since the usage of NPs as a carrier provides enhanced solubility and biodistribution, they are also highlighted with their potential in gene therapies by carrying nucleic acids [55]. Gold NPs draw a notable interest in gene therapies due to their presence in the area. Their low toxicity capacity, high uptake efficiency, and capacity enhance their potential for gene silencing and transfection [56]. Different gold NPs, such as positively charged, nanorod, or small-sized, have been coated and utilized for efficient DNA and RNA delivery (Figure 5) [57]. Moreover, the surface charge density of gold NPs is tuned to enhance the gene delivery application. It is possible to adjust the charge density of gold NPs through amino acid modification, thus increasing the efficiency of nucleic acid delivery [58]. This is why nucleic acid delivery of gold NPs is applied in various approaches like other gold NP-based delivery systems. Stimuli-responsive delivery of nucleic acids, such as GSH-mediated release or via light irradiation, are promising alternatives in this manner [59]. Similar to the previous section, along with the later section that discusses the anticancer activity of gold NPs, gold NPs are also used in cancer treatment through their efficient nucleic acid delivery. Their capability to be modified with wide-ranging probes makes them preferred to deliver RNA silencers to tumors [60]. Therefore, gold NPs are utilized to carry vectors and induce desired intracellular activity following cellular uptake for multiple purposes.

##### DNA

DNA is a promising target in gold NP-based nucleic acid delivery. Most of the time, the DNA is bound to the surface of the NP with various types of interactions. Attachment of the DNA to gold NPs can be mediated with sulfur (S) (more preferable) and nitrogen (N) bonds or with the charge interactions through the surface charge of the particle [62]. In addition, high amounts of DNA can bind into gold NPs with great bond strength [63]. This feature is commonly highlighted in the modification of gold NPs with DNA, which is highly used in different types of applications, including drug delivery [64]. Still, there is a notable amount of research efforts that utilize this feature in nucleic acid (DNA) delivery applications.

As an example, PEG-functionalized positively charged gold NPs were used to deliver nonviral vectors (three types that range between 4 and 40 kpb) to various cell lines (HeLa and Hek293t cells) [65]. Based on the cell viability results, doses below 50 μg/mL for HeLa and below 25 μg/mL for Hek293t cells were used. The evaluation of the transfection efficiency of the plasmid DNA was observed in fluorescence microscopy and compared to the control group. As a result, up to a 20% increase in the fluorescent intensity was observed. Later on, the potential of the gene therapy was evaluated with expression of herpes virus thymidine kinase through treatment with the prodrug ganciclovir. Compared to commercial formulations, the cell viability of the HeLa cells was significantly lower due to the efficient gene delivery by the gold NPs. Another method was deployed to co-deliver DNA and siRNA with multi-layered degradable polymer coatings onto gold NPs [66]. High doses of DNA (200 to 2400 ng) and siRNA (160 to 240) was used in layered gold NPs. Both siRNA-mediated knockdown and DNA-mediated expressions were determined during the experiments. Up to 25% knockdown was achieved in siRNA delivery, as the highest rate was obtained on days 6–7 with the 240 ng loading. The fluorescence images revealed the DNA expressions of both, including one and two nucleic acid DNA-layered formulations. Two-layered gold NPs, without siRNAs, showed the highest transfection ratio of 28%.

One particular study used gold NPs to deliver donor DNA and clustered regularly interspaced short palindromic repeats (CRISPR)–associated protein 9 (Cas9) ribonucleoprotein (RNP) to induce DNA repair *in vivo* [67]. The induction of homology-directed repair by the complex was observed in human embryonic kidney cells. Before the test, the induction of the DNA repair, encapsulation efficiency (61.5%), and enzymatic activity (preserved with encapsulation) were observed. In *in vitro* conditions, the DNA repair of 11.3% of the total cells was induced by the treatment of CRISPR-gold NPs. Additionally, the rate of the DNA repair reached its maximum at a concentration of 8 μg/mL, while exceeding this value caused cellular cytotoxicity by the CRISPR-gold NPs. The *in vitro* study extended to dendritic and primary myoblast cells, along with multiple types of stem cells. The CRISPR-gold NPs managed to target all the used types of cells and exhibited a DNA repair ratio between 3 and 4% through simultaneous delivery of the Cas9 protein, the donor DNA, and guide RNA. The gene editing significance of the system was also demonstrated in a reporter mouse model. At last, CRISPR-gold NPs are intramuscularly injected into mice for the possibility of correcting dystrophin mutation. The results showed that the particles successfully corrected the dystrophin gene (5.4%) and restored the dystrophin protein expression. The treatment without the gold NPs showed an 18-fold lower (0.3%) correction rate. The treatment improved the strength and muscle function of the mice with extremely minimal off-target genomic damage (between 0.005 and 0.2%). These promising results not only extend the application of the CRISPR-Cas9 system but also show that gold NPs can also be applied to complex systems as an efficient carrier.

##### RNA

Usually, RNA molecules are bound to the surface of gold NPs with thiol or electrostatic interactions [68]. However, various types of RNAs (especially siRNAs) can be bound on gold NP surfaces with additional interactions: electrostatic interactions (the layer-by-layer approach is included), DNA hybridization, and crosslinking [69]. Most of the time, the siRNAs are bound with gold NPs to enhance their stability and delivery efficiency. This approach has been used for various purposes.

For instance, an *in vitro* study was conducted to improve the delivery of the siRNAs to inhibit dengue virus infection [70]. Against four serotypes of dengue virus, antiviral siRNAs were tested in Vero cells. Except for serotype 1, the antiviral activity of the siRNAs was demonstrated against the dengue virus. Later on, these siRNAs were modified with gold NPs by the layer-by-layer (electrostatic) approach and delivered to Vero cells. Transmission electron microscopy (TEM) images confirmed that gold NPs were successful in the delivery of siRNAs into the cell. Finally, the complex was tested for potential inhibition of dengue virus infection at both pre- and post-infection phases. Both viral propagation and virus replication were inhibited by the gold NP-siRNA complex. The complex exhibited efficient inhibition by demonstrating up to 15-fold higher inhibition than the control group. Additionally, significant protection of siRNAs by gold NPs was shown, along with their protected activity after treatment of RNases.

Gene silencing is commonly preferred in RNA delivery with gold NPs. Conde *et al*. demonstrated *in vitro* and *in vivo* gene silencing through the delivery of siRNA with gold NPs for RNA interference (RNAi) application [71]. A primary gene, c-myc protooncogene, was chosen as a target for RNAi and targeted in HeLa cells, in freshwater polyps, and in *in vivo* mouse models for gene silencing. The attachment of siRNAs to gold NPs was mediated with ionic (with the negative charge of siRNA) and covalent (thiol–gold bond) approaches. TEM images showed the accumulation of gold NP conjugates in the cytoplasm of the cells. To detect the efficiency of the gene silencing, the luciferase gene was used as a reporter. As expected, there was a significant reduction in luciferase activity by the siRNA activity from 110–100% (control groups) to 50–25% (depending on the attachment approach). The same results were not observed when the gold NPs were bound with non-related siRNAs, indicating the selectivity of the gene silencing. *In vivo* models were not different in terms of gene silencing efficiency. Both approaches that mediated the siRNA bonding significantly reduced the *Hydrac-myc* expression levels to less than 50% (the covalent approach was superior compared to the ionic). Similar results were also obtained in a mouse model, as the covalent approach showed significant inhibition of mouse-myc expression levels (to nearly 30%) compared to the ionic approach (nearly 60%). These findings not only represent the potential of gold NPs in therapeutic applications but also highlight the importance of the chosen approach in gold NP-based RNA delivery complexes.

The approach for releasing the RNAs is as important as the approach for binding the RNAs on the NP surface. Stimuli-responsive delivery is a common alternative that is used in gold NP-based RNA delivery. The light-irradiation method was used on gold nanorods in the treatment of pancreatic tumors through the co-delivery of siRNA (for K-Ras gene silencing) and doxorubicin [72]. Several types of gold NP samples were used during the experiment, including separately coated doxorubicin and siRNAs. Fluorescent images of Panc-1 cells confirmed that the efficient cellular uptake and the transfection efficiency was found to be higher than 83%. Each sample (the ones that included doxorubicin and siRNA) managed to decrease the mRNA and protein levels and effectively inhibited K-Ras expression. This, later on, was explained by the blocked proliferation of the Panc-1 cells through S-cell cycle arrest by gold nanorod-mediated delivery. Furthermore, the release profiles were observed under the 665 nm light irradiation. A burst release of doxorubicin (90%) was observed under 20 min with light irradiation; meanwhile, the drug release reached 80% after exceeding 16 h. A similar profile was also observed for siRNA release, with more than 60% of the RNAs released in under 20 min. Without light irradiation, the same released amounts were reached after the sixth hour. Light-irradiated formulations showed the highest tumor suppression *in vivo* as well. Another stimuli-responsive RNA delivery was mediated with capped gold nanorods but to deliver small hairpin RNAs, not siRNAs [73]. A significant tumor gene silencing was observed with the gold nanorod–RNA complex. Both *in vivo* and *in vitro* experiments showed the enhanced RNA release by the glutathione triggering.

Gold NPs possess great potential in nucleic acid delivery by their favorable surface modification. Depending on their shape and other characteristics, methods to mediate delivery systems show diversity, indicating the presence of wide-ranging approaches. With slight differences, both gene expression and silencing can be induced with the gold NP models. Based on the discussed studies and the current literature, gold NPs are also utilized in more complex systems and different applications.

#### 3.1.3. Protein Delivery

**Figure 6:**
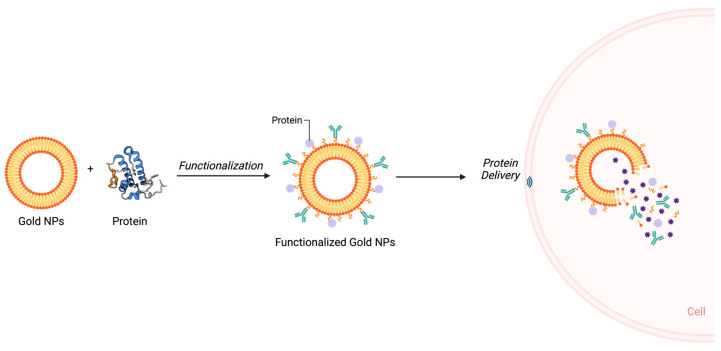
Representation of protein delivery mechanism of gold NPs. By altering their surfaces with ligands, polymers, or linkers, gold NPs may be made to bind particular proteins. This increases their circu-lation time and stops enzymatic breakdown. Through endocytosis, gold NPs enable cellular ab-sorption and release protein cargo inside cells. Therapeutic applications benefit from surface changes that improve targeting to certain tissues or cell types [75].

Similar to nucleic acids, many types of proteins are attached to the gold NP surface for cell-targeted delivery purposes. Protein-coated gold NPs possess enhanced internalization with directed targeting and modulated functions (Figure 6) [74].

Most commonly, protein/peptide-based functionalization of gold NPs is utilized for cancer cell targeting or to increase the efficiency of the drug delivery capability of the particle [76]. Nevertheless, many studies used gold NPs as a nanocarrier to delivery therapeutic enzymes and peptides for various applications, including but not limited to anticancer, biosensing, and neurodegenerative diseases [77]. It is possible that gold NPs can be utilized in many major application areas with their enhanced delivery characteristics. In this manner, we have evaluated a few studies that conjugate various types of proteins onto gold NPs. Additional recent studies are given in Table 1.

Ghosh *et al*. demonstrated the intracellular delivery of an enzyme (β-galactosidase), which is incapable of passing through cellular membranes, with peptide-coated gold NPs as a protein transporter [78]. The modified gold NP–protein complex was treated with HeLa cells and then administered X-gal to observe a color change upon enzymatic hydrolysis. Compared to the control group (sole treatment of the enzyme), a significant color change was observed in the gold NP–protein complex group, approximately 98% at the highest protein concentration. Another study used DNA aptamer-conjugated gold NPs for *in vivo* delivery of functional proteins [79]. The researchers loaded His-tagged proteins on gold NPs and performed both *in vitro* and *in vivo* experiments. A significant internalization of the protein through gold NP carrier was demonstrated in the *in vitro* study. *In vivo* animal models demonstrated significant antitumor activity by the carrier protein (BIM protein) by tumor-targeted delivery.

One study included gold NPs in a nanocomposite, including manganese ferrite NPs, for efficient delivery of milk protein bovine lactoferrin (bLf) for antifungal therapeutic applications [80]. To test the antifungal activity, bLf-loaded nanocomposite was tested on *Saccharomyces Cerevisiae* along with solo bLf and unloaded nanocomposite treatments. The cell viability results indicated that both solo bLf and loaded nanocomposite significantly inhibited fungal cells, showing the efficient release of the protein from the NPs. Lactoferrin exhibits significant antimicrobial activity, including antifungal activity [81,82]. A novel, site-directed nanosystem that can mediate the transport of this protein might possess new therapeutic applications for both Lf and other milk proteins. As an example, in addition to Lf, both β-Lactoglobulin and α-Lactoglobulin [83,84] were conjugated with gold NPs, highlighting their potential in targeted drug delivery.

Concerning peptide delivery, an *in vivo* study was conducted to deliver a peptide (ovalbumin) vaccine to induce an antitumor response with gold NPs [85]. Mice injected with gold NP–peptide complex show a significant increase in interferon-gamma-producing splenocytes; meanwhile, control groups or sole peptide-treated mice barely show any increase. Later on, these groups’ tumor sizes and areas (mm^2^) were investigated. At day 20, both control and only-peptide-treated groups exhibited the highest tumor size, while gold NP-delivered peptide groups did not show any increase in tumor size. Tumor areas were also boosted between days 6 and 8 for control and solely peptide-treated groups. The survival rate of the mice was also similar, which was 100% in the gold NP-delivered group. As a result, gold NP-mediated peptide delivery managed to inhibit tumor growth in mice models and preserved the survival rate at the highest levels. Similar to other molecules that have been discussed, peptides are conjugated onto gold NPs for delivery of other molecules, such as DNA. Niu *et al*. demonstrated the utilization of peptide-conjugated cationic gold NPs for gene delivery to reverse the progression of melanoma [86]. The results indicate a significant internalization, high transfection, and efficient skin penetration for possible gold NP-based topical gene therapy.

When the gold NP-based protein delivery approaches are considered, certain types of proteins and protein-based structures are highlighted, including but not limited to enzymes, peptides, and structural proteins. Since gold NPs can be conjugated with various types of proteins, their potential in protein delivery should not be overlooked.

#### 3.1.4. Antibiotic Delivery

Antibiotic delivery is another promising application of gold NPs, especially in dealing with antimicrobial resistance (AMR). Recently, gold NPs have been highlighted as a potential drug delivery agent to combat AMR, with much recent research existing in the current literature [87]. There are certain approaches in which gold NPs are used for antibacterial applications, such as photothermal therapies, various types of ligand conjugations, or pristine gold NPs [88].

A study showed the delivery of amino-glycosidic antibiotics (four types) with protein-capped (bovine serum albumin), stabilized gold NPs [89]. Each type of antibiotic (streptomycin, neomycin, gentamicin, and kanamycin) was tested on three types of bacteria for its antibacterial activity, with and without the gold NP carrier. Each antibiotic–gold NP conjugate demonstrated enhanced antibacterial activity, from 4.7% to 33.3% for *Escherichia coli (E. coli)*, from 21.0% to 50.0% for *P. aeruginosa*, and from 18.1% to 66.66% for *Staphylococcus aureus (S. aureus)*. Similar research was conducted on multi-drug-resistant (MDR) bacteria [90]. Gold NP-mediated and solo administrations of the antibiotics were compared on MDR bacteria *Klebsiella pneumoniae* (*K. pneumoniae*), *S. aureus*, and *E. coli*). Solo treatment of gold NPs and antibiotics did not show any zone of inhibition. Meanwhile, all antibiotics showed a zone of inhibition between 7.2 and 10.7 mm when delivered with gold NPs. Minimum inhibitory concentration (MIC) and minimum bactericidal concentration (MBC) values were significantly reduced with gold NP delivery, along with the bacterial survival rate.

In addition to conjugation, Meeker *et al*. showed biofilm inhibition by photothermal and antibiotic co-treatment through the incorporation of antibiotics onto polydopamine-coated gold nanocages [91]. The major finding during the experiment is the reveal of the insufficient antibacterial activity of the PTT gold nanocages without antibiotic loading. The nanocages successfully reduced the bacterial cell numbers, yet after 24 h, the viable cells started to reach back to the control levels. However, this was not the case when the PTT was applied to antibiotic-loaded gold nanocages, which indicated significant cell-targeted antibacterial activity by the complex. Another study showed the impact of antibiotic-loaded gold NPs on MIC values against various bacteria isolates [92]. According to the size of the gold NPs, the smallest-size particles (35 nm) exhibited the highest loading efficiency. The antibacterial tests showed that using gold NPs as an antibiotic carrier decreased the MIC values up to 3- and 4-fold.

The findings demonstrate that both conjugation and loading of antibiotics to types of gold NPs possess significant potential in MDR treatment. The antibiotic-based approach represents one example of the gold NP-based antimicrobial activity. Many studies highlight the antibacterial activity of various types of gold NPs with MDR bacteria strains. Even without antibiotic conjugation, gold NPs are still effective against MDR bacteria through modification with other biomolecules. For instance, a study showed the antibacterial activity of gold NPs against MDR bacteria with carbohydrate coating [93]. The study also demonstrated the *in vivo* activity of the particles and indicated their potential in wound healing through MDR-based antibacterial activity. 

### 3.2. Anticancer

We have discussed the applications of anticancer drug delivery with gold NPs. However, many studies indicate the direct anticancer activity of the gold NPs (Table 2). It was suggested that gold NPs can initiate anticancer activity through ROS formation, which can lead to DNA and mitochondrial damage, along with caspase activation (apoptosis) [94]. It is important to note that the mechanism behind the anticancer activity of gold NPs can vary depending on their charge density. Gold NPs with a high positive charge density can induce mitochondrial abnormalities and alter intracellular Ca^2+^ concentrations, which is disadvantageous to cancer cells, whereas negatively charged particles are less effective [95]. Additionally, compared to positively charged particles, highly hydrophobic particles can promote oxidative stress rather than directly influencing mitochondria or Ca^2+^ levels [95]. Similar to many inorganic metal NPs, the concentration of the gold NPs in treatment possesses a primary factor. In the anticancer activity of gold NPs, this factor is also extremely valid. As an example, an *in vitro* study showed the anticancer activity of green-gold NPs against lung and liver cancer cells (Hep-G2 and A549) [96]. The anticancer test was performed depending on the used NP concentrations (1 μg, 10 μg, 25 μg, 50 μg, and 100 μg). After the gold NP concentration exceeded 25 μg, observable cell viability changes were seen in both cell lines. Subsequently, the highest concentration showed the most significant and respectable changes in the cell viability percentage.

As a result, green synthesis of the NPs, especially metal NPs since they possess wide-ranging activity and applications with a great risk of toxicity, are currently the most highlighted synthesis method in the literature [97]. In the anticancer activity of gold NPs, green synthesis is also highly considered to reduce any potential cytotoxicity against normal cells [94].

**Table 2:** Recent anticancer applications of gold NPs.

Application	Synthesis Method	Properties	Results	Reference
Anticancer activity against osteosarcoma	Green synthesis using *Phormidesmis communis* strain AB_11_10	Average size of 9.6 ± 4.3 nm. Size between 4 and 20 nm (chemically synthesized). Quasi-spherical and triangular morphology. SPR peak at 524.5 nm.	- Significant cytotoxicity against MG-63 and SAOS-2 cell lines with 50% inhibition at concentrations 297.5 and 15.5 µg/mL, respectively. - Chemically synthesized gold NPs inhibited 50% of cells at concentrations of 72 and 62 µg/mL, respectively. - Green-synthesized particles showed significant specificity against SAOS-2 cells.	[98]
Anticancer activity against pancreatic cell lines	Chemical synthesis	Mean sizes of 83 ± 20 nm (coated with hyaluronic and oleic acids) and 49 ± 12 nm (coated with bombesin peptides). Spherical morphology.	- Significant anticancer activity against BxPC-3 tumor cells with combined treatment of radiation therapy.	[99]
Determination of anticancer and antioxidant properties of green-synthesized NPs	Green synthesis from *Coleus scutellarioides* (L.) Benth leaves	Average size of 40.10 nm. Spherical morphology. SPR band at 532 nm.	- At maximum concentration (120 µg/mL), DPPH scavenging activity is determined by 38.07%. - Significant cytotoxicity against MDA-MB-231 cell line (IC50 at 36.10 µg/mL).	[100]
Anticancer effect on hepatic carcinoma through immunoregulation	Green synthesis from polygahatous polysaccharides	Average sizes of 10–14 nm (green-NP) and 30–34 nm (NP). Spherical morphology.	- Induction of TNF-α and IL-12p70 levels (*in vitro*). - Increased body weights of mice (decreased when NPs administered with adriamycin). - Increased serum TNF-α levels and CD4+/CD8+ lymphocyte ratios and decreased serum IL-10 levels (*in vivo*). - Increased tumor inhibition rate and decreased tumor growth.	[101]
Determination of anticancer property	Green synthesis using the seed extracts of *Momordica cymbalaria*	Average size of 38 nm. Spherical morphology.	- Decreased cell viability of lung cancer lines to nearly 20% levels through ROS synthesis and apoptosis induction. - Up to 58% inhibition of ROS synthesis. - Antibacterial activity against *Klebsiella pneumoniae*. - Capability to protect proteins and DNA from oxidative damage.	[102]
Anticancer and anti-plasmodial activity	Green synthesis from multiple types of leaf extracts	Size between 13.8 and 25.1 nm (depending on the extract). Polydisperse and spherical morphology.	- Significant inhibition of cancer growth up to 96% at the highest concentration, 50 μg/mL (IC 50 at 8.253 μg/mL). - Effective antiplasmodial activity with high selectivity.	[103]
Determination of anticancer property	Green synthesis from *Chrysothemis pulchella* leaf extracts	Average size of 14.7 nm. Spherical morphology. Absorption band at 527 nm.	- Significant cytotoxicity against HEK 293 and HeLa cells with IC50 values of 34.5 and 54.05 µg, respectively. - Strong antimicrobial activity.	[104]
Anticarcinogenic activity	Chemical synthesis	Average size of 14 nm. Spherical morphology. Absorbance peak at 520 nm.	- Significant reduction in cell viabilities of MCF7 and A549 cancer cell lines to 31.25% and 28.13% at the highest concentration of 100 µM, respectively. - Significant increase in TNF-α levels and apoptosis levels.	[105]
Anticancer activity against lymphoma cells	Green synthesis from *Moringa Oleifera* leaf extract	Size ranging from 6 to 18 nm. Spherical, trigonal, and hexagonal morphologies.	- Significant reduction in cell viability of Dalton’s lymphoma to approximately 30% at the highest concentration of 150 μg/mL (IC50 at 75 ± 2.31 μg/mL). - Induction of apoptosis through nuclear fragmentation, diffuse chromatin condensation, and increased apoptosis protein expression. - Increased ROS levels by 68.41% at the highest concentration. - Loss of mitochondrial membrane potential by 50.21%. - Cell cycle arrest at G2/M phase by increase of 35.66%.	[106]

Another primary mechanism behind the anticancer activity of the gold NP is its influence on the apoptotic pathway. Research showed that green-synthesized gold NPs inhibited cancer cell proliferation through the apoptotic pathway [107]. Starting from 2.5 μg to 25 μg, various doses of gold NPs were used in the anticancer test. Similar to previously discussed results, increased concentration significantly decreased cell viability compared to lower doses. To determine the effect of gold NPs on apoptotic proteins, levels of caspase 3, caspase 8, and caspase 9 were observed. The colorimetric assay revealed that the increases in the concentration and levels of these proteins were directly proportional. Change in the caspase proteins indicates the induced apoptosis of the cells by the gold NPs.

One final gold NP-based approach that is used in anticancer applications is cancer immunotherapy. Along with the PTT and drug delivery, these discussed approaches in anticancer activity are widely used to trigger tumor-specific immune responses, including wide-ranging immunotherapeutic agents/genes on many models [108]. Ong *et al*. demonstrated the combined treatment of cancer immunotherapy and PTT with gold NP-doped silica NPs [109]. The nanocomposite was internalized in bone-marrow-derived dendritic cells, and their activation was investigated in terms of immunotherapy. The activated cells demonstrated a significant increase in CD11c and CD86 percentages, along with increased TNF-α and IL-12 secretion. As a result, the desired inhibition of tumor growth and survival rate in mice were observed. Another study also showed the significant induction of TNF-α, IL-6, and granulocyte-colony stimulating factor expression by gold NP-based immunotherapy application [110]. However, one important feature that needs to be highlighted in the study is the impact of the particle size. The peak induction was only observed in the small-sized (15 nm) NPs, while larger particles (30 nm and 80 nm) could not manage to cause induction.

Overall, without involving the PTT, gold NPs can prevent tumor growth and inhibit cancer cell growth with several approaches, either directly or indirectly. When looking into the anticancer application of gold NPs, excluding the anticancer-related drug and peptide delivery studies, most types of experiments are predominantly found *in vitro*. Since there is not a certain optimum concentration for treatments and various factors that influence the toxicity capacity of the gold NPs, especially size, the application of *in vivo* models might be hindered. Still, considering the current research, the study of gold NPs in anticancer applications should preserve their importance for further findings with their great potential.

### 3.3. Photothermal Therapy Applications

PTT is a powerful method that generates heat energy when receiving light energy (such as NIR), which is a commonly used approach for killing tumor cells [111]. Since gold NPs possess unique optical characteristics, such as absorbing and scattering light in the visible regions, they are used in PTT to enhance the anticancer application (Table 3) [112]. The LSPR of gold NPs is one of the main factors that significantly alters the PTT application. Depending on the size and the temperature, the LSPR property of the gold NPs can be greatly influenced, possibly affecting the efficiency of PTT [113]. In addition to size, the shape of the gold NPs changes the light absorption at various wavelengths, altering their application depending on the area [114]. Moreover, the current literature widely combines gold NP-based PTT with other types of therapies for precise and efficient anticancer application.

#### 3.3.1. Gold NP-Based PTT for Anticancer Application

PTT treatment is quite common in gold NP-based anticancer studies. Gold NP-mediated PTT treatment can induce several anticancer mechanisms, including the induction of necrosis (through structural changes in protein and lipids) and apoptosis (increased gene expression) of cancer cells [126]. Most recently, this approach is combined with various treatments to increase the efficiency and overcome sensitivities during the treatments. However, some findings indicate the efficient anticancer application of solo PTT treatment. Most importantly, these studies are crucial to determine the impact of the physical properties of gold NPs, such as size and shape, in PTT-based anticancer applications, since they greatly affect cell penetration and heat generation.

Yang *et al*. showed the influence of the shape on gold NP-based photothermal cancer therapy [127]. Three types of gold NP shapes were tested during the experiment: nanospheres, nanorods, and nanostars. Under NIR light irradiation, gold nanostars showed the most efficient photothermal conversion (46.2%) compared to the other two types (21.6% and 20.4%). All types of NPs were tested with and without NIR light irradiation in a toxicity test. Even at the highest concentration (200 μg/mL), only gold nanorods managed to show lower cell toxicity, which was approximately 80%. On the other hand, all types of gold NPs demonstrated significant cytotoxicity, which was extended to nearly 30% by the gold nanostars at the highest concentrations. The *in vitro* cytotoxicity test indicated the enhanced anticancer activity of gold NPs with PTT, with a great dependence on their shapes. Fluorescence images of the cells significantly visualized the decreased cell numbers by NIR light irradiation, especially in gold nanostars. Thanks to gold NPs’ unique LSPR effect, all cells are exposed to local hyperthermia by light irradiation, resulting in a promising potential for PTT in cancer therapy.

Depciuch *et al*. demonstrated the influence of the size in photothermal conversion efficiency using spherical gold NPs [128]. Between 8 and 16 nm, various sizes of gold NPs were tested with two different irradiation levels, 650 and 808 nm. The researchers showed that the smallest gold NPs exhibited the most efficient photothermal conversion, up to 70%. Most visible morphological and chemical alterations in *in vitro* experiments were observed by the smallest particle as well. Even though the small-sized gold NPs showed the most reduction in cell viability tests, the difference between the particles was not significant. The authors highlighted that this might be because of the difference between the size of the particles, which are quite small.

**Figure 7:**
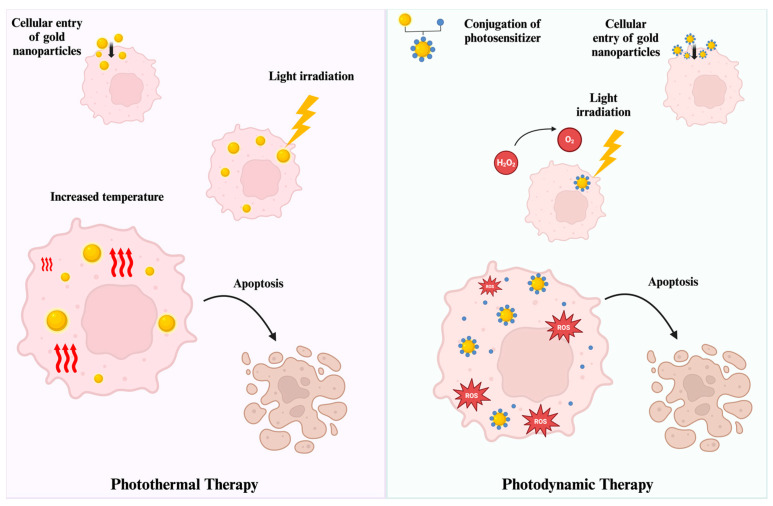
Gold NP-based photothermal and photodynamic therapy in anticancer application [52,129].

As highlighted in the previous sections, the physical properties of the gold NPs, especially LSPR and size, impact the utility of gold NP-based PTT. Solo treatment of gold NP-based PTT is quite effective in anticancer applications (Figure 7). Since the PTT-based anticancer activity of gold NPs is widely discussed in the current literature, we have evaluated gold NP-based combined therapies using PTT for anticancer applications.

#### 3.3.2. Gold NP-Based PTT with CRISPR-Cas9 System

Photothermal therapy of gold NPs is utilized in wide-ranging applications. As highlighted previously, anticancer and tumor-targeting applications heavily use PTT with gold NPs. Recently, gold NP-mediated PTT has been under discussion for application with the CRISPR-Cas9 system in tumor therapy [130]. It was highlighted that the photothermal effect of gold NPs can be used for efficient DNA release by excitation with laser irradiation and combined with the CRISPR-Cas9 system as a photothermal release agent. In addition, since PTT can lead to increased tumor death through NIR-responsive nanomaterials, a gold NP-included CRISPR-Cas9 system is also an alternative for anticancer research [131].

To give an example, a gold nanocomposite, with various plasmon resonances, was used to deliver a CRISPR-Cas9 system for synergistic gene–photothermal therapy [132]. A multi-branched gold nano octopus was loaded with CRISPR-Cas9 RNP and coated with PEG-folic acid. The synthesized nanostructure demonstrated 99.7% internalization into tumor cells. The gold NP-CRISPR system under irradiation showed the most significant reduction in cell viability, demonstrating lower than 20% with strong antitumor activity. The accumulation of the system was similar in the *in vivo* model, showing high fluorescence intensity. The normal and single PTT-treated groups exhibited limited antitumor activity. However, most importantly, synergistic PTT treatment with NIR irradiation showed the most significant antitumor activity by inhibiting tumor growth. The synergistic PTT group also demonstrated 26.7% gene disruption, indicating the desired gene editing performance of the system.

A similar synergistic application of the CRISPR-Cas9 system was mediated with gold nanorods (derived from cancer cell membrane) to target cancer cells [133]. With a great targeting and cellular uptake ratio, both the solo treatments of PTT and gene therapy and their combination significantly decreased cell viability and high apoptosis levels *in vitro*. Combined treatment demonstrated an approximately 40% higher apoptosis rate compared to the solo therapy of both groups. Gene editing efficiency was also estimated as 33%, while it was 23% when PTT was not applied. The antitumor activity and tumor inhibition were the greatest in the combined therapy group (with NIR irradiation) in the *in vivo* model. The most impactful results were observed in the decreased relative tumor volume.

For the past few years, nanotechnology-based CRISPR-Cas9 delivery systems have been extremely highlighted. Many types of NPs, including gold NPs (especially nanorods), have been tested with plasmid-based, mRNA-based, and RNP-based CRISPR-Cas9 [134]. Gold NPs possess multiple characteristics related to their increased potential as an alternative for PTT applications [135]. Taking these into consideration, gold NP-based CRISPR-Cas9 systems may create a significant influence on PTT applications.

#### 3.3.3. Gold NP-Based PTT Combined with Immunotherapy

Similar to gene therapy, PTT is also combined with immunotherapy for anticancer treatments. Tumor cells can attempt to avoid the immune system by increasing the secretion of certain immunosuppressive cytokines (such as interleukin-10 and tumor growth factor-beta), upregulation of programmed death ligand 1 (PD-L1), and downregulation of MHC class I molecules [136]. It is thought that adding immunotherapy to combined therapy applications can reverse these strategies by including tumor-specific T cells and checkpoint inhibitors (such as PD-L1, which is described below) in the therapy [52]. As highlighted in this section, this approach proved the superior efficiency of combined therapy compared to solo treatment of each therapy.

A study demonstrated the combined therapy of PTT and immunotherapy against tumor cells with dendric cell-derived gold NPs [137]. Both *in vivo* and *in vitro* experiments demonstrated significant antitumor activity of gold NPs through laser irradiation (non-NIR treatment did not manage to decrease cell viability or tumor growth in mice). Gold NP treatment with NIR almost completely inhibited the tumor growth by showing a 96.7% suppression rate. A significant increase in the numbers of T cells and cytokines were observed with gold NP treatment. The increase in the numbers was almost doubled when particles were treated with NIR. This combination was also highlighted in recently published research. For example, T-cell-carrier gold NPs demonstrated enhanced antitumor activity when compared to the monotherapy of both approaches [138].

Another study highlighted the enhanced antitumor response of gold nanostars when PTT was combined with dendritic-cell-based immunotherapy and anti-PD-L1 immune checkpoint blockade therapy in a mouse model [139]. The gold nanostar-mediated PTT treatment with solo antibody administration negatively affected the antitumor response by increasing PD-L1 expression and tumor-to-muscle ratio in mice. The separate treatment of both therapies limitedly affected the tumor growth, and antibody treatment demonstrated antitumor activity with lesser efficiency. Yet, combined therapy with antibody treatment significantly extended the survival rate of mice to 60%, along with the least tumor volume values, while other groups did not survive to day 30. The PD-L1 expression was approximately reduced by 90% and reversed the non-sensitive effect of PTT therapy, indicating the significance of the combined therapy.

Such research demonstrates that gold NP-based PTT treatments can be inefficient in anticancer applications under certain conditions. Even if it demonstrates sufficient activity, the enhanced results from combined therapy are nonexpendable. To achieve the most efficient and reliable therapy approach for gold NP-based therapies in anticancer studies, these combinations should be further investigated.

### 3.4. Photodynamic Therapy Applications

PDT is a method used to initiate photon energy transfer through NIR that is mediated by photosensitizers [140]. Upon exposure to NIR light, a photosensitizer initiates the electron transfer, thus generating ROS that create a cytotoxic environment for cells [141]. This mechanism is extremely beneficial in the applications that aim to initiate the destruction of targeted cells, including bacteria. PDT can initiate the destruction of cancer cells through the activation of photosynthesizers that are specifically internalized into tumors [142]. Cellular uptake is crucial in the determination of the efficiency of PDT due to the main mechanism of the photosynthesizer in light exposure. This is why NPs with strong optical properties that are capable of carrying these agents hold significance in PDT treatments, especially in cancer treatment [143].

The delivery of photosensitizers with NPs can be performed through various methods such as surface-binding and encapsulation, along with the unbound co-application and only NP application as the photosensitizer itself [144]. Considering the high utility of gold NPs in anticancer research, gold NP-based PDT applications can increase the photosensitizers inside cancer cells (Figure 7). This is why the usage of photosensitizer-conjugated gold NPs is extremely prestigious in PDT-based anticancer applications (Table 4) [129]. A similar approach is also used in antimicrobial applications. Many metal NPs can be used in PDT to initiate bacterial cell death through the NIR of the PDT agent [145]. Thanks to the strong LSPR property, different types of gold NPs can be used in PDT to initiate cellular death of bacteria [146].

#### 3.4.1. Gold Nanoparticle-Based PDT in Antimicrobial Applications

Methylene blue is a common photosensitizer used in PDT in antibacterial and anticancer applications [155]. In studies that utilize gold NP-based PDT for antibacterial applications, methylene blue is usually used as the photosensitizer as well. Moreover, the addition of gold NPs in PDT, in which methylene blue is the photosensitizer, can enhance the efficiency of the therapy in antibacterial application. The enhanced antibacterial application of PDT was demonstrated in research using biogenic gold NPs to decrease methylene blue photobleaching [156]. The reduction in the cell viability of bacteria (*S. aureus* and *E. coli*) with methylene blue treatment reached 61% and 45% at the highest concentration (250 mg/L), respectively. The addition of light irradiation to methylene blue and gold NP groups increased the cellular death up to 99.87%. At the highest light intensity, the results were more significant, showing 99.96% cellular death in the gold NP–methylene blue group. The kinetic profile of methylene blue revealed that the involvement of gold NPs significantly lowered photobleaching and slowed down the photo-fading process. The results showed that gold NP-based PDT not only is effective in antibacterial applications but also can enhance the overall efficiency of PDT when methylene blue is used as the photosensitizer. An *in vitro* study demonstrated the antibacterial and antibiofilm application of methylene blue-conjugated gold NPs through PDT on *Streptococcus mutanss* [157]. The enhanced antibacterial activity from PDT was observed in minimum inhibitory and bactericidal concentrations, which were the lowest. Even though it required a higher inhibitory concentration (250 µg/mL), gold NPs showed inhibition in sole treatment. Under 490 and 570 nm wavelengths, gold NP-based PDT significantly prevented biofilm formation compared to other groups.

In addition to bacteria, some studies aim to utilize gold NP-based PDT in some types of fungi and viruses. Based on this, a study combined the PDT and PTT, with methylene blue being the photosensitizer, to demonstrate the enhanced antifungal activity with gold NPs through the addition of P-123 copolymer [158]. An *in vitro* susceptibility test on *Candida albicans* showed that gold NPs alone did not induce notable antifungal activity with solo treatment of PTT, which included only green LED light. However, the addition of P-123 and methylene blue, the combination of both red and green LED light, demonstrated the reduction in the desired concentrations in fungal cells. Yet, it was highlighted that heat generation from gold NPs could negatively affect cellular death, potentially by the reflected radiation from methylene blue to gold NPs. Still, the researchers suggested that increased concentrations of gold NPs and light could enhance the treatment. An additional *in vitro* test was performed on *E. coli* and *S. aureus* bacteria, which was more effective compared to the fungal test in terms of gold NP efficiency. Another thing that needs to be mentioned is the ineffectiveness of P-123 copolymer treatment against bacteria, with and without gold NPs, indicating the crucial contribution of PDT in gold NP-based antimicrobial treatment.

Some studies have involved gold NP-based PDT in fungal research, especially on *C. Albicans* [159,160]. Even though recent studies involved methylene blue-based PDT against *C. albicans* with other types of structures, such as micelles [161] and nanomaterials [162], the up-to-date research studies are quite deficient in terms of gold NPs. The inadequacy of studies is more severe for antiviral applications.

Considering the efficiency of gold NP-based therapies, PDT-based applications can be an alternative in certain antimicrobial applications. The most important potential is enhanced PDT efficiency by adding gold NPs in the treatment. Hereupon gold NPs might be a leading alternative in PDT-based applications, at least within the current applications of gold NPs. Even though antimicrobial activity is not the strongest part of gold NPs’ application, there is a probability that this insufficiency can be improved with PDT and combined therapies.

#### 3.4.2. Gold Nanoparticle-Based PDT in Cancer Applications

The delivery of photosensitizers with gold NPs is commonly utilized in *in vitro* anticancer research. As previously discussed, the cellular uptake of photosensitizers is a critical factor in determining the efficacy of PDT-based cancer treatments. Given the drug delivery capabilities of gold NPs, photosensitizer-conjugated gold NPs are emerging as promising candidates for the future of PDT.

An *in vitro* example of PDT application of gold NPs for anticancer treatment was conducted on MCF-7 breast cancer cells [163]. Hypericin, a photosensitizer, was conjugated with gold NPs to improve its internalization for PDT. The intracellular localization of hypericin was significantly enhanced with gold NP conjugation, showing higher intensity and a two-fold increase in uptake, particularly in lysosomal and mitochondrial regions. The gold NP-treated group exhibited significant changes in cell morphology post-PDT, as highlighted by increased LDH levels, reduced ATP levels, and increased apoptosis (both early and late stages), indicating the effectiveness of PDT with gold NPs.

This strategy is extremely advantageous for improving the efficacy of PDT. However, in some cases, the treatment’s effectiveness may not meet the desired outcomes. To address this, one common approach in PDT-based anticancer applications using gold NPs is the combination with PTT. Given the effectiveness of PTT in targeting cancer cells, a combined PDT-PTT therapy holds considerable potential in anticancer applications and overcomes the limitations of either therapy alone. Since gold NPs exhibit unique optical properties and serve as transporters for delivery agents, systems involving these particles are highly significant in anticancer research. For example, one study highlighted the combination of PDT and PTT using surface-modified gold NPs conjugated with photosensitizers [164]. Dendrimer-modified gold NPs demonstrated the highest fluorescence intensity, indicating significant levels of intracellular ROS. Combined treatment of PTT and PDT resulted in high internalization of the gold NPs into cancer cells. The involvement of both PDT and PTT agents showed a nearly 3-fold impact on cell viability compared to non-irradiated groups. The effectiveness of the combined therapy was further demonstrated in *in vivo* results, achieving up to 88% tumor suppression.

Another example of combined therapy was applied to osteosarcoma, where gold nanotriangles were used to enhance tumor targeting and deliver photosensitive drugs as nanoprobes [165]. The internalization of the gold nanoprobes was observed near the mitochondria of U2OS cells. Detection of the singlet oxygen molecules generated from PDT was conducted using a laser confocal microscope, confirming the successful delivery of the photosensitive drug. The nanoprobe group showed higher signal levels than other groups thanks to the efficient cellular uptake and protection of the drug during the delivery. Under light irradiation (808 nm), separate treatments of photosensitizer (for PDT) and gold nanotriangles (for PTT) managed to induce apoptosis in 31.7% and 30.7% of total cells, respectively. As expected, combined treatment nearly doubled the apoptosis induction by demonstrating 64.9% of total cellular death. Similar observations were seen in the *in vivo* experiment, where separate treatments demonstrated survival rates of 40% and 60%, while the nanoprobe group demonstrated a 100% survival rate with maximum tumor growth inhibition.

Similarly designed studies indicate the enhancement of the anticancer activity in combined therapies. Gold NPs are highly effective in absorbing or scattering the irradiated light, along with the delivery of the photosensitizer for improved cytotoxicity. Both *in vivo* and *in vitro* results show significant improvements compared to solo applications of the therapies. As with drug delivery strategies, gold NPs could serve as the foundation for future synergistic systems involving PTT and PDT in anticancer research.

### 3.5. Bioimaging and Biosensor Applications

Gold NPs offer a significant advantage in sensing applications, as the oscillation of electrons under irradiation results in a detectable color change [166]. There are various types of gold NP-based biosensors, such as optical, electrochemical, and piezoelectric, each exhibiting significant sensitivity due to gold NP properties [167]. Gold NPs are commonly utilized in the development of electrochemical sensors. Thanks to their high surface area and unique electrochemical properties, both gold-based nanomaterials and gold NPs are used mainly for the detection of various biomolecules, including nucleic acid and proteins [168]. Various studies in the current literature highlighted the role of gold NPs for detection purposes with electrochemical-based sensors [169,170]. Similarly, piezoelectric biosensors are a promising alternative that expand biosensing-related applications. Numerous studies in recent years have utilized gold NPs in the development of piezoelectric biosensors with high efficiency, enabling the detection of specific biomolecules [171] and microorganisms [172]. In addition to these types of sensors, gold NPs are predominantly used in LSPR and SERS-based sensors due to their strong electrochemical properties, optimal conductivity, and highly resonant particle plasmons (Table 5) [173].

#### 3.5.1. Gold NP-Included SERS Sensors

Gold NPs are highly emphasized in both *in vitro* and *in vivo* bioimaging studies due to their SERS, which allows for extreme sensitivity, precise determination of chemical bonds, and a wide range of surface modification possibilities [166]. The physical properties, especially the shape and arrangement of the particles, are key features that impact the application of gold NPs in SERS sensors. The efficiency of these sensors can be enhanced by optimizing the physical properties of the gold NPs.

Huang *et al*. compared the effects of a planar array and a bridge array of gold NPs on the DNA hybridization efficiency of SERS sensors [185]. There was an approximately 3-fold difference in the normalized fluorescence intensity between the planar and bridge arrays, with saturation times of 180 and 60 min, respectively. The normalized Raman ratio and fluorescence intensity were nearly 5-fold higher in the bridge array after 3 h. Overall, the bridge array exhibited a 15-fold higher efficiency compared to the planar array. The bridge array was further tested for microRNA-21 detection, where the gold NP-based DNA sensor successfully detected microRNA-21 concentrations in both HeLa and MCF-7 cells.

Certain shapes of gold NPs are specifically preferred due to their unique properties that enhance the activity of SERS sensors. Gold nanostars are known for their ability to increase electromagnetic field intensity, leading to a significant increase in light absorption [186]. Additionally, the structure of gold nanostars, particularly their sharp surface tips, further enhances SERS activity. This was highlighted in a study demonstrating the compatibility of gold nanostars for colloidal SERS detection of probe molecules [187]. The sharp branches of gold nanostars enhanced the SERS activity compared to those with rougher surfaces.

Gold NP-based SERS sensors have been tested in various fields for multiple purposes. It would be challenging to include and discuss all of these applications in detail. However, a few recent studies in the current literature are evaluated below to briefly demonstrate the latest applications of gold NP-based SERS sensors.

SERS-based sensors are in heavy demand and possess potential in current agricultural applications for precise detection of chemicals to ensure food safety [188]. There are certain recent applications in which gold NPs are used for that reason, especially for pesticide detection.

Several recent studies highlight the modified gold NPs’ efficient detection of pesticide residues with SERS-based approaches. For instance, Duan *et al*. developed a sensitive SERS-based detection of pesticide residues (chlorpyrifos) with buck paper-modified gold NPs [189]. The synthesized gold NP–buck paper SERS substrate demonstrated significant SERS activity and reproducibility. Moreover, it demonstrated high SERS intensity in the direct detection of chlorpyrifos, showing a 0.937 linear correlation coefficient. At last, the pesticide detection was confirmed on peach samples, showing a recovery rate between 94.2 and 115.5%. Another study designed a hydrophobic SERS sensor for pesticide residue detection through gold NPs that are decorated on silicon nanorod arrays [190]. The designed sensor demonstrated a significant LSPR effect that was induced by the gold NPs. The improvement led to an enhancement of the electric field on the nanorods’ surface. A very low standard deviation between 4% and 6% was also a notable finding during the pesticide residue detection.

Gold NPs are also used for simultaneous pesticide detection, especially in juice samples for future food safety research. Based on this, a research study used gold–silver NPs as a SERS substrate for the detection of multiple pesticide residues (thiram and acetamiprid) in juice samples simultaneously [191]. The SERS method showed the intensity of both residues in the juice samples with notable peaks in Raman shifts. Moreover, the addition of both residues at the same time was also detected by the SERS method, indicating the simultaneous detection capability of the system. The accuracy of the SERS method was observed at the recovery rate of thiram and acetamiprid, which was between 90.38 and 113.24% and between 89.86 and 122.12%, respectively.

Gold NPs are usually used as SERS tags through surface modifications with Raman reporters and further coated with target ligands if necessary [192]. As a result, depending on the physical properties, gold NPs can enhance Raman scattering and brightness of SERS tags for strong SERS responses during detection.

You *et al*. demonstrated the SERS-based detection of *E. coli* with starch magnetic beads coated with gold NPs with SERS tags [193]. The induction of local enhancement of the electromagnetic field was observed specifically when the SERS tags were able to interact with the target bacteria. The gold NP-based SERS system successfully detected the bacteria even at the lowest concentration (1 CFU/mL) with a high specificity.

Another example for gold NP-conjugated tags was conducted for dopamine detection, where the particles were coupled with nanolaminate plasmonic crystals [194]. Gold NPs, conjugated with Raman tags, were combined with plasmonic crystals for dual recognition of the dopamine. Both the Raman tag in the gold NPs and *N*-hydroxysuccinimide ester functionalized plasmonic crystals sense and bind into dopamine, constituting an “off” and “on” system in signal levels depending on the presence of dopamine. The Raman intensity of modified plasmonic crystals was compared with label-free SERS sensing, where there was a 1.3-fold difference on behalf of the crystals. Once again, the Raman intensity was strongly enhanced by the involvement of the gold NPs, showing the potential of the dual recognition of the system. Based on the “on” and “off” system, a quantitative determination of dopamine was also conducted. Digital SERS sensing results demonstrated enhanced dopamine specificity, high reproducibility (93.7% recovery rate), and accurate determination capability of the system.

#### 3.5.2. Gold NP-Based LSPR Sensors

LSPR-based sensors take advantage of the shift in gold NP’s LSPR wavelengths since they are heavily influenced by interparticle interaction, environment, and surface modifications, especially the size and shape of the NP [195]. Based on this, LSPR-based sensors determine the change in the local index of refraction that occurs on the surface of the NPs, making a sensitive detection of the molecule [196].

Compared to SERS sensors, where gold nanostars are highlighted, gold nanorods possess unique optical features for LSPR sensors. They exhibit extreme sensitivity to longitudinal plasmon wavelengths, with a high extinction coefficient that leads to observable bright colors, and they enable plasmon band shifts dependent on distance, allowing for additional assays in applications [197].

Certain studies use gold NPs in LSPR sensors for hormone detection. As an example, Gao *et al*. recently used a gold NP-based composite fiber sensor, utilizing both SPR and LSPR, for dopamine detection [198]. The SPR performance measurement revealed that the gold NP included a composite that coupled both SPR and LSPR, indicating a significant enhancement of SPR signals, detection sensitivity, biomolecular absorption capacity, and surface electric field intensity. The authors indicate that compared to other dopamine fiber optic sensors, they demonstrate the lowest detection limit and highest detection range for dopamine mediated by fiber optic sensors. The sensor demonstrated a noteworthy linear response in desirable concentrations that give potential for dopamine detection in practical solutions. Another recent study combined the LSPR absorption of gold–silver NPs and fluorescence intensity of carbon dots as a rapid and sensitive sensor for dopamine detection [199]. The sensor showed significant specificity towards dopamine by generating notable signals in the existence of common substances from human serum. When tested on human serum, the sensor had a low standard deviation (lower than 5%) and a high recovery rate between 92.51 and 104.79%, indicating their potential in biomedical analysis. The gold NP-based LSPR sensors were also used for the detection of other hormones, such as serotonin [200] and anti-Müllerian hormone [201].

Another common application of LSPR-based gold NPs is for the detection of biomarkers for diagnosis purposes. An LSPR label-free biosensor was used for dengue diagnosis with gold nanospheres [202]. Experiments on the optical properties of gold NPs and refractive index-based sensings showed that the size of the NPs heavily influences the sensor’s efficiency. Based on the tested sizes (2.5, 30, and 50 nm), 30 nm gold NPs were used for dengue antigen detection with the most efficient LSPR properties. The gold NPs demonstrated molecular identification at very low concentrations (1.50 nM), highlighting the system’s strong sensing ability. Another LSPR-based biosensor was used for the detection of alpha-synuclein biomarkers using mercaptoundecanoic acid-capped gold nanorods [203]. The recovery rates and coefficient of variations of synuclein were determined between 97.30 and 118.65% and between 1.114 and 11.903%, respectively. The lower limit of detection (11 pM) and high selectivity of the platform on alpha-synuclein oligomers indicate another potential of the gold NPs in LSPR-based sensors in diagnosis applications.

### 3.6. Other Biological Applications

Up to this point, we have covered most of the predominant applications in gold NP research. NP characteristics and unique optical properties generally form the basis of their applications. However, it is worth mentioning that gold NPs are employed in various biological applications for multiple purposes.

#### 3.6.1. Antimicrobial Activity

**Figure 8:**
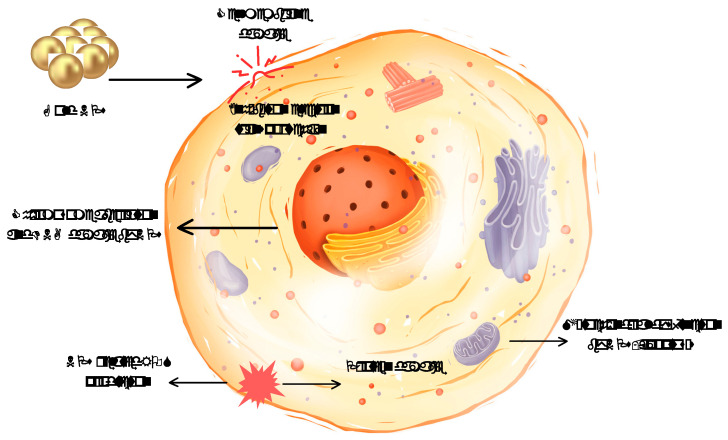
Antibacterial activity of gold NPs by multiple mechanisms [209].

Various types of gold NPs, including nanostars, nanorods, and nanoclusters, have demonstrated significant antibacterial activity through several main mechanisms: ROS-mediated DNA and membrane damage, cell wall disruption, and structural disturbance, including PTT-based approaches (Figure 8) [204]. In addition to shape, surface modifications are commonly used in antibacterial applications [205], especially with other types of metals and organic compounds [206]. Many antibacterial applications of gold NPs utilize their surface chemistry, which is easily manipulated based on the intended target [207]. Synthesis methods are also under investigation. Gold NPs derived from various plant extracts have demonstrated strong antibacterial activity, showing considerable zones of inhibition [208].

When considering the anticancer studies in Table 2, it is evident that most studies aim to determine multiple properties of gold NPs (especially if the synthesis method is green) rather than directly investigate the particles’ antibacterial or anticancer activities. We have discussed various antimicrobial applications of gold NPs in this review. In many cases, the study of anticancer and antibacterial activities leverages characteristic properties like optical behavior and surface modification. Despite similarities with other metal NPs, gold NPs can, in specific cases, exhibit significant antimicrobial activity, which can vary based on the physicochemical properties of the particle.

Dheyab *et al*. demonstrated the potential antibacterial activity of gold NPs depending on the synthesis method [210]. In the comparative study, gold NPs were synthesized using both sonochemical and reduction methods. The synthesized particles exhibited spherical morphology, with average diameters of 18.5 and 20 nm, respectively. The zeta potential of NPs from the sonochemical method was determined as −48 mV, which was higher and indicated greater stability compared to the reduction method at −21 mV. Later on, the antibacterial efficiency of these two gold NPs was tested on *S. aureus* using inhibition zones. Highly stable, sonochemical-synthesized gold NPs demonstrated an 11 mm inhibition zone at the lowest concentration (20 µg/mL), while gold NPs synthesized by the reduction method showed no inhibition zone. At the highest concentration (80 µg/mL), there was a 2 mm difference between the two methods, with the sonochemical gold NPs demonstrating the higher value. The potential mechanism of action behind the antibacterial activity was briefly discussed, involving the penetration of cell membranes, leading to various interactions with DNA and other intracellular components.

As highlighted, numerous studies have employed green methods to synthesize gold NPs and evaluate their antibacterial capacity, along with other properties. One study demonstrated the anticancer, antifungal, antibacterial, and catalytic properties of spherical gold NPs synthesized from *Pistacia vera* hull extracts [211]. The antibacterial assays revealed that gold NPs exhibit significant potential against various bacterial strains, with Gram-positive bacteria being more susceptible to the particles. The most significant MIC value was 0.5 µg/mL for *E. faecalis*, which was close to the value of ciprofloxacin, 0.21 µg/mL. Additionally, gold NPs were effective against MDR strains as well, showing a range of MIC values between 34.3 and 137.5 µg/mL. The antifungal tests also showed positive results, but only for two strains of *Candida albicans* (IFRC1873 and IFRC1874) with 550 and 137 µg/mL, respectively. The other three types of fungi were resistant to the gold NPs with no measurable MIC values. The catalytic and anticancer activities of the particles were also discussed.

Another study demonstrated various properties of green-synthesized gold NPs from *Trichoderma saturnisporum*, along with silver NPs [212]. There was an expected difference in the MIC values of silver NPs and gold NPs, with values differing 2- to 4-fold (120/250 to 500/1000 µg/mL). Although silver NPs required lower concentrations to demonstrate antibacterial activity, gold NPs exhibited higher antibiofilm activity, ranging from 59.4% to 93.6% depending on the administered concentration (15.62–250 µg/mL). In contrast, silver NPs exhibited antibiofilm activity ranging from 39.36% to 57.5%. Moreover, it was shown that gold NPs demonstrated slightly higher antioxidant and anticancer activities compared to silver NPs except at the highest concentration of 1000 µg/mL. Numerous other studies highlight the multiple properties of green-synthesized gold NPs, including their antibacterial activity [213].

Although fewer in number, some recent studies have also investigated the antifungal and antiviral activities of gold NPs. One study demonstrated the antifungal activity of green-synthesized gold NPs from *Allium sativum* against Candida species [214]. Significant toxicity from gold NPs was observed with increased levels of LDH and ROS, leading to cellular disruption-mediated death. Another study also demonstrated antifungal activity of the green-synthesized gold NPs from an extracellular extract of the fungus *Schizophyllum commune*, along with anticancer and delivery capacity [215]. The gold NPs produced inhibition zones of 2.3 and 2.7 cm against *Trichoderma* sp. and *Aspergillus flavus*, respectively. Additionally, antibiotic conjugation increased these values to 2.5 cm and 2.9 cm, respectively. Moreover, scanning electron microscopy (SEM) images revealed damage on the spore walls caused by gold NPs. Increased ROS levels in lung carcinoma A549 cells were also evaluated. Similar examples are provided in Table 6.

Antiviral activity of gold NPs against influenza virus was demonstrated in an experiment, along with their anticancer activity [238]. A hemagglutination inhibition assay (HAI) was conducted to evaluate the antiviral efficiency of gold NPs. Three phases of infection were tested for antiviral activity: pre-incubation, post-incubation, and current incubation. The results showed that gold NPs were most effective when introduced to the virus during pre-incubation. Current incubation and post-incubation also demonstrated antiviral activity, though to a significantly lesser degree. The tissue culture infectious dose 50 (TCID50) and PCR values of the virus were lowest during the pre-incubation with gold NPs. Another *in vitro* study demonstrated antiviral activity of green-synthesized gold NPs from the blue-green algal strain *Spirulina platensis* against the herpes simplex (HSV-1) virus [239]. During the study, the antiviral activity of gold NPs was compared to that of green-synthesized silver NPs. The reduction rate of gold NPs was 42.75%, while silver NPs exhibited a higher rate of 49.23%. A potential mechanism for the antiviral activity of gold NPs against HSV-1 was proposed [240]. The study revealed that pre-treatment with gold NPs reduced the cytopathic effect of HSV-1 and decreased viral load up to 100-fold, depending on time, concentration, and size (with smaller particles being more effective). The authors hypothesized that absorption of gold NPs onto the virion may block viral attachment, thus preventing cellular entry. More recent experiments have primarily focused on the conjugation of gold NPs, as shown in Table 6.

#### 3.6.2. Wound Healing

Considering the wide-ranging utilization of NPs in wound healing applications, gold NPs also stand as a potential agent in the area. Considering their more tolerable toxicity levels and accumulated research on their green synthesis, they are regularly studied for their potential [241]. Their antibacterial activity and ROS scavenging capacity, including their significant research background with various therapies and modifications, can be enhanced in levels that are required in wound management [242]. The general mechanisms behind the induction of wound healing by gold NPs, which are highlighted in various research studies, are the inhibition of bacterial growth (most likely through modifications and/or combined therapies), adjusting the levels of immune response, supporting the formation of fibroblasts, and regulating ROS levels [243,244].

Antibacterial activity is typically the primary characteristic desired in materials used for wound dressings. In addition to promoting wound healing and providing antioxidant activity, antibacterial activity is crucial to inhibiting the growth of bacteria commonly found in the early stages of many wounds [245]. Research has shown the antibacterial effects of gold NPs (synthesized with *Lavandula angustifolia* essential oil) on MDR wound-causing bacteria [246]. Treatment with only gold NPs or *Lavandula angustifolia* demonstrated MIC values of 256 and 128 µg/mL against *Proteus mirabilis*, respectively. However, conjugating the essential oil onto gold NPs reduced the MIC value to 8 µg/mL. The MBEC values matched the MIC values for both compounds, and the conjugated nanostructure reduced it to 16 µg/mL. Additionally, both the conjugated nanostructure and gold NPs showed visible wound healing efficiencies of 96.78% and 28.91% and wound closure rates of 70.74% and approximately 40%, respectively. We have detailed that the modification and conjugation of gold NPs are among the most impactful factors tailoring their application. This example, considering the visible differences in results, further indicates the importance of these factors in gold NP applications.

Another study utilized biogenic gold NPs (derived from the sericin protein of *Bombyx mori* silk cocoon) to investigate wound healing activity, along with antioxidant and antibacterial potential [247]. Contrary to expectations, low concentrations of gold NPs (5 and 10 µg/mL) showed the highest wound closure efficiencies of 70.96% and 69.76%, respectively, compared to the 25 µg/mL concentration, which was approximately 60%. However, the cell viability tests showed values higher than 94% in all concentrations. The antibacterial activity was tested on four types of foodborne pathogens, with the highest concentration showing the most significant MIC (25 µg/mL) and MBC (50 µg/mL) values, except for *E. coli*, where the values were doubled.

#### 3.6.3. Anti-Inflammatory

There have been several mechanisms that potentially comprise the anti-inflammatory activity of gold NPs: reduction of oxidative stress, leukocyte adhesion, pro-inflammatory cytokines, and induction of macrophage polarization [248]. Various *in vivo* and *in vitro* studies in the past demonstrated the anti-inflammatory activity of gold NPs through reduction of cytokine levels [249].

For example, the green synthesis of gold NPs from citrus peel extract demonstrated enhanced anti-inflammatory activity when synthesized using ultrasound [250]. Gold NPs were tested in two forms: with ultrasound-assisted synthesis and without it. Both types of gold NPs were shown to decrease nitric oxide (NO) synthesis, with IC50 values of 82.91 and 157.71 μg/mL, respectively. The authors suggested that the enhanced anti-inflammatory activity was due to the reduced size of the gold NPs from ultrasound treatment. Additionally, dose-dependent inhibition of cyclooxygenase-2 and inducible nitric oxide synthase mRNA expression was demonstrated. Another study demonstrated the enhanced anti-inflammatory activity of green-synthesized gold NPs from *Diospyros kaki* fruit extracts against skin inflammatory diseases [251]. Significant inhibition of interleukin 6 and 8 (IL-6 and IL-8) secretion and activation-regulated chemokine was achieved with pre-treatment of gold NPs at concentrations of 100 and 200 µg/mL. Additionally, mRNA expressions of interleukins and chemokines were also observed. Moreover, gold NPs downregulated MAPKs/NF-κB signaling pathways. Further discussion on the skin moisturizing activity of gold NPs was also included.

#### 3.6.4. Antidiabetic Activity

In addition to the previously mentioned biological activities, there are some studies that highlight the antidiabetic potential of gold NPs. One frequently highlighted activity is the antidiabetic effect of gold NPs. One study demonstrated enhanced antidiabetic activity of gold NPs synthesized from *Physalis minima*, alongside the evaluation of their antioxidant and antimicrobial activities [252]. The antidiabetic potential of gold NPs was demonstrated through an alpha-amylase inhibition assay, where enzyme inhibition ranged from 90 to 93%. Another study used green-synthesized gold NPs from *Moringa oleifera* leaves, showing alpha-amylase inhibition with an IC50 value of 130 µg/mL [253]. Given the suggested potential of metal NPs as novel inhibitors of alpha-amylase enzymes [254], it is unsurprising that gold NPs demonstrate similar activity in some studies.

## 4. Toxicity

Gold NPs are attracting attention because of their distinctive physicochemical and visual characteristics. They are being investigated in biomedical domains, specifically for localized thermal ablation of neoplastic cells following light irradiation.

Nonetheless, despite their significant potential applications in biological, environmental, and industrial fields, there is scant evidence regarding the short- and long-term health effects on organisms and the broader environment. Synthesized NPs can traverse the body without eliciting immune system rejection, attributable to their surface charges and diminutive size. The health concerns stem from their physicochemical features; nevertheless, there is insufficient evidence regarding their health impacts and an absence of regulatory safety requirements for their toxicities. The impact of these NPs on future health outcomes continues to be cause for concern. Despite numerous studies indicating the low toxicity of gold NPs relative to other metal-based NPs, their extensive application necessitates an assessment of their consequences in consumer products. Gold is regarded as innocuous owing to its chemical inertness, yet it is a desirable material because of its nanoscale characteristics, which include programmable sizes, easy manufacture and modification, and strong optical capabilities [255]. Their non-toxic properties offer a major benefit in delicate applications including medication administration, biosensing, and cancer treatment. Unlike many other metals, which emit potentially harmful ions, gold is chemically stable and compatible with biological systems. However, a number of variables, including dose, delivery methods, surface changes, and particle size, can affect how biocompatible gold NPs are. There is variation in the methods and findings of research on gold NPs; some contend they are innocuous, while others contest this. Understanding their possible uses requires a thorough evaluation of toxicities and safety issues [256]. For example, smaller particles may cause inflammation or oxidative stress at larger dosages and are more easily able to pass through cellular membranes. By covering gold NPs with biocompatible substances such as PEG, possible toxicity is decreased and tolerance is increased in the body. Although gold NPs are typically regarded as non-toxic biomaterials, careful testing is necessary to guarantee their safe usage in each particular application. Because of variations in size, shape, surface charge, and coating material, gold NPs interact with biomolecules, cells, and tissues in diverse ways. Contradictory results have been obtained from research conducted under a variety of settings, including variations in size, shape, surface changes, cell lines, and tests. This might be due to differences in toxicity testing, cell lines, and NP properties. To obtain a thorough judgment, more research is required [257].

**Figure 9:**
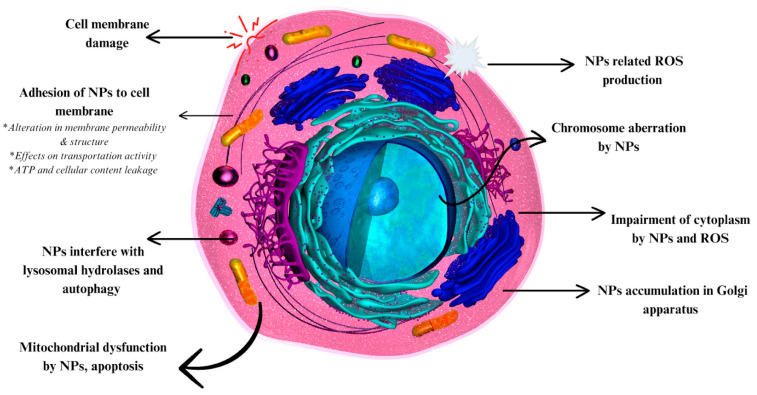
Potential toxicity mechanisms of gold NPs [257,258].

A variety of potential mechanisms, including genotoxicity, ROS production, mitochondrial damage, cell death pathways, toxic material leakage, the interaction of endocrine disruption with molecules, and changes in cell shape, are taken into consideration when evaluating the toxicity of gold NPs (Figure 9) [258]. Together with these potential mechanisms, there are several factors that affect the toxicity of gold NP mechanisms. The dimensions of gold NPs, measuring fewer than 100 nm, are critical for their biological function and toxicity. These NPs can traverse biological barriers via self-assembly mechanisms, accessing previously unreachable locations. Nonetheless, their diminutive dimensions may cause cellular harm, resulting in cytotoxicity, genotoxicity, and inflammatory reactions, presenting possible health hazards [259]. On the other hand, the morphology of gold NPs considerably influences their interaction with biological systems, as various forms such as rods, spheres, and cubes affect their cellular uptake and distribution. Rod-shaped NPs have distinct biodistribution patterns, influencing their toxicity and usefulness in biomedical applications [260].

The physicochemical characteristics of gold NPs, which internalize them within cells and render them more poisonous than larger particles, are the main cause of their interactions with biological systems. Because of their high surface area-to-volume ratio, this emphasizes how crucial their size is in biomedical systems [261]. Moreover, the overall charge of gold NPs can significantly influence their toxicity capacity. Due to the increased cellular interactions of NPs with high charge density, the surface charge of the particles should be tailored according to the intended application. As an example, a previous study highlighted that positively charged gold NPs can accumulate in skin tissue up to 2–6 times higher than negatively charged NPs [262]. The charge density can also affect the mechanism. While unmodified gold NPs may induce necrosis under certain conditions, highly charged particles can induce p53 expression, thereby triggering cell death via apoptosis [263]. Nonetheless, a developing paradigm in green toxicology places a strong emphasis on creating gold NPs that are safer by design [264].

It is thought that gold NPs accumulate in the spleen and liver, possibly leading to more harm. Gold nanorods were delivered subcutaneously into mice, resulting in oxidative damage to the tissue. To evaluate their dispersion and accumulation in organs, researchers have carried out many investigations. Lopez-Chaves *et al*. (2018) tested the distribution and impact of gold NPs on HT-29 and HepG2 cells as well as Wistar rats. Traces were discovered in the kidney, spleen, liver, gut, urine, and feces. Particle size affected excretion and biodistribution pathways, with the smallest particles inflicting more harm [265]. It is conceivable because, according to Schmid *et al*., ultra-small gold NPs have distinct biodistribution and better circulation times than larger gold NPs [266].

According to Lipka *et al*., rats that were treated intratracheally showed a buildup of PEG-coated gold NPs in their liver and spleen [267]. Furthermore, the liver of mice underwent acute inflammation and apoptosis due to PEG-coated gold NPs. Similarly, a size-dependent ROS-induced cytotoxicity was generated by the PEG-coated gold NPs. Additionally, accumulations of 42.5 and 61.2 nm in size are typically found in the liver and spleen. The authors discovered that gold NPs’ *in vitro* toxicity depends on their size and dosage, with smaller sizes and greater concentrations causing cytotoxicity and more cell damage via the generation of ROS. Size also affects biodistribution, which shows components of buildup and clearance [268].

Kadhim *et al*. (2021) investigated the toxicity of spherical gold NPs on embryonic fibroblast cells (REF) both *in vitro* and *in vivo*. REF cells were treated with gold NP doses of 1, 5, and 10 μg/mL for 48 h, followed by intraperitoneal administration at 100 μg/Kg for 28 days. Toxicity was determined to be merely 10% at the maximum gold NP dosage, with even reduced values noted at concentrations of 1 and 5 μg/mL. The NPs did not influence the body weight of mice, hence affirming the safety of gold NP application at low quantities [269]. Lasagna-Reeves *et al*. (2010) investigated the efficacy of gold NPs in drug delivery and illness diagnostics using 12-week-old mice. Various amounts of gold NPs were delivered, with the maximum concentration detected in the liver. The research indicated that the uptake and absorption of gold NPs predominantly transpire in tissues, with the proportion of stored gold diminishing as the dosage increased, implying effective elimination from the organism. The research assessed the nephrotoxicity of gold NP in mice by analyzing levels of urea nitrogen, uric acid, and creatinine. The results indicated no significant variations in metabolites or inflammation, and no tissue damage was seen in any regions of the kidneys, liver, spleen, brain, or lungs [270].

A thorough toxicological analysis of gold NPs on mammalian cell lines was presented by Chuang *et al*. The results showed that gold NPs significantly alter the expression of 436 genes and protein functions linked to cell cycle progression and apoptosis [271]. Gold NPs disrupt the expression of 19 genes in human fetal lung fibroblasts according to a similar result by Ng *et al*. [272].

A comprehensive research background is essential for clinical translation of gold NPs. Even though they show less toxicity potential compared to their counterparts, gold NPs suffer from deficiency in terms of clinical applications. Noteworthy clinical trials conducted in recent years suggest the potential use of gold NPs. This review includes two of these trials to provide an overview of the current clinical status of gold NPs. More of these applications in the past years, including PTT-based therapies, drug delivery systems, and therapeutic activity, were included and discussed in a recent review [273].

One study conducted peptide delivery with gold NPs with microinjection as an immunotherapy in type 1 diabetes [274]. Three doses of conjugated gold NPs were given with a 4-week gap between each dose. The follow-up phase extended up to week 52, with only four participants completing the trial. Safety evaluations indicated a reduction in gold concentrations in blood serum, first observed after the first week following the third dose. The reduction became more significant by week 20, with gold being completely cleared from the blood by week 52. No adverse side effects or hypersensitivities directly related to the injected particles were reported. However, all participants experienced delayed local skin reactions that gradually faded over a period of up to 24 months.

Another similar trial used small interfering RNA-conjugated gold NPs for treating patients with recurrent glioblastoma [275]. Eight patients were intravenously administered conjugated gold NPs at a very low dose concentration (0.04 mg/kg). The researchers reported no grade 4 or 5 adverse effects during the treatment. However, two grade 3 adverse effects were reported during the trial, though these were considered possible rather than directly related to the treatment. Analysis from two of the tumor samples revealed that 41% and 81% of the administered gold concentration accumulated in the tumor tissue. Analysis of accumulated RNA in the tumor region highlighted the following findings in the trial: caspase-3 activation, increased p53 protein expression, and downregulation of Bcl2L12 protein.

Examination of the overall data indicates that gold NPs tend to accumulate in the body over the long term after treatment. However, given the unique characteristics and promising results of gold NPs, future trials may demonstrate that these disadvantages can be overcome by the substantial benefits gold NPs uniquely offer [276].

The toxic characteristics of gold NPs require an extensive safety evaluation. Through the integration of sophisticated analytical techniques, toxicological research, and concepts of green toxicology, researchers and policymakers may guarantee the responsible production and utilization of gold NPs, thereby fostering sustainable applications in various industries, safeguarding public health, and preserving the environment.

## 5. Future Trends

A review of papers including “gold nanoparticles” in the title and published between 2019 and 2024 (Figure 2) reveals a slight decrease in the number of papers each year. However, when specific applications are searched in titles, 41% of the total published papers on gold nanoparticles are related to these applications. Given the predominance of property-based and synthesis-based gold NP research in recent years, the fact that nearly half of the published papers focus on these applications underscores the significance of gold NP applications in current trends. The total number of published papers in these areas shows either an increase or stability each year compared to past years except for the most recent year. However, it is worth noting that this year’s total published articles may show a decrease compared to last year.

Antioxidant activity is predominantly included in studies investigating the biological activity of green-synthesized gold NPs. Considering the other biological activities tested alongside antioxidant activity, especially anticancer and antimicrobial activities, it can be assumed that most antioxidant studies in the last five years overlap with other types of studies, since they are often included in the same research. One specific reason for that is the majority of the recent research aims to determine the biological properties of novel, green-synthesized gold NPs from various biosources. This is why antioxidant studies are shown as the fourth most studied application, with a 9% share among the discussed applications of gold NPs. The same cases apply to some other types of applications as well. For instance, some antimicrobial and anticancer studies are also involved in drug delivery applications. Considering the wide-ranging PDT and PTT-based anticancer research, we did not conduct a search based solely on the “anticancer” term in the titles. To draw a clear conclusion and provide precise data about the distribution of gold NP applications within the overall research, these factors should also be considered.

Still, applications derived from the most unique feature of gold NPs are not affected by this overlap. Biosensor, bioimaging, PDT, and PTT-based applications are all investigated solely based on the optical properties of gold NPs (a slight deviation can still be observed when combined PDT/PTT treatments and biosensor applications are considered). These four applications comprise approximately 40% of the studies in the sections discussed in this review. Considering the involvement of carrier capabilities, the majority of studies in the last five years have heavily utilized the optical properties of gold NPs. This factor clearly indicates the extraordinary potential of gold NPs in diagnostics and medicine applications. Considering the shift of green synthesis methods and toxicity studies, overcoming the current limitations in gold NP research can enhance the clinical viability in the area.

**Figure 10:**
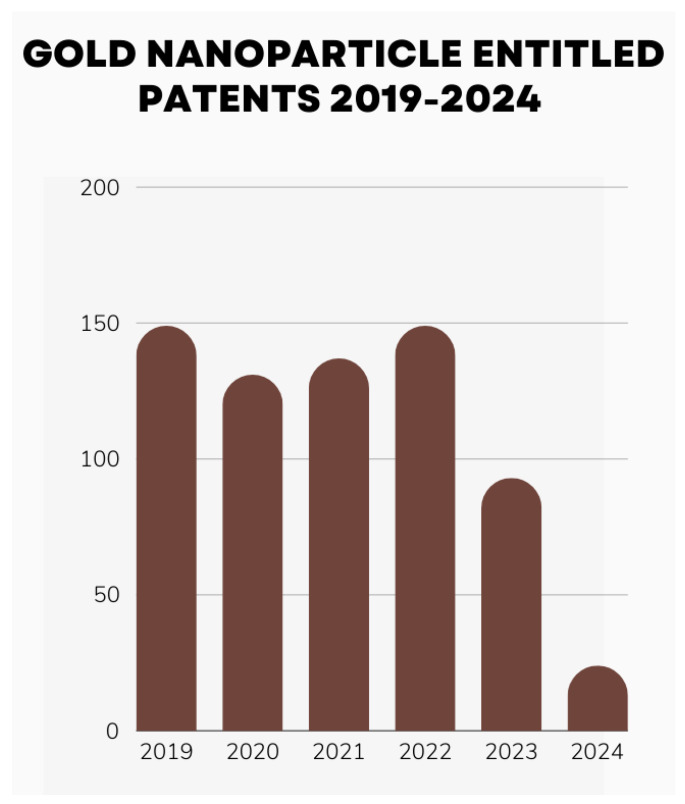
Number of registered patents containing “Gold Nanoparticle” in their title in the last five years [277].

Recently, gold NPs’ frequent utilization in a wide range of applications, from optical to antibacterial, positioned them as one of the most studied nanomaterials. Based on information provided from Google Patents, approximately 700 patents including “gold nanoparticle” in their title have been registered in the last five years (Figure 10).

Although there has been a slight decrease in the number of published documents between 2019 and 2022 (Figure 2), the number of created patents remained at a constant level with nearly 150 patents per year. This implies a strong interest in advancing NP technologies, including the development of green synthesis approaches, drug delivery systems, and cancer therapies, where gold NPs are regarded as the main focus. However, data indicate a decrease starting from 2023, which might be attributed to the growing focus on green synthesis approaches. Lately, researchers are conducting experiments with a wide array of alternative materials for the production of gold NPs. In most of these studies, the main focus is the modification of input materials, where core synthesis methods remain unchanged. This consequently leads to a situation where fewer new patent protections are required, as the fundamental process is adapted to different materials rather than being completely reinvented.

Despite stabilized improvements in patent registrations and the literature publications, significant challenges in the biomedical applications of gold NPs persist. Even though gold NPs are highlighted as a biocompatible nanomaterial in various biomedical applications [278], they still possess bioavailability concerns that affect their clinical applications, as discussed in the previous section. Although green synthesis methods hold significant potential to address this challenge, their synthesis efficiency and large-scale production remain limited [279,280]. Deficiencies in clinical trials negatively impact patent registrations. Advancements in synthesis methods that ensure the stability and optimize the physicochemical characteristics of the particles could pave the way for future developments in personalized medicine [281].

Even though there is a decrease in the number of registered patents, high demand towards gold NPs persists. There are many studies in the industrial area focusing on gold NP utilization in development of biosensors and environmental remediation approaches. Hence, establishing production methods that are more cost-effective and scalable would be crucial to address the restoration of patent activity in this area.

## 6. Conclusions

Gold NPs have been highlighted in the current literature with their unique optical properties. They exhibit significant carrier capability, potential antimicrobial activity with alterations of surface chemistry, and magnificent anticancer activity through multiple approaches. Gold NPs offer a lot of potential in biological applications because of their distinct physicochemical characteristics, especially LSPR. Numerous functionalizing agents, including polymers, surfactants, ligands, dendrimers, medications, DNA, RNA, proteins, peptides, and oligonucleotides, can be coupled with these NPs, which are easily synthesized, modifiable, biocompatible, and non-toxic. Thanks to their surface plasmon absorption, gold NPs also have exceptional optical qualities, which makes them valuable for sensing, imaging, and labeling. They show promise for cancer detection, imaging, diagnostics, and therapeutic applications in gene therapy and other disorders since they are biocompatible and readily conjugated with biomaterials. Applications for miniature devices, such as nanobarcodes, are also of interest regarding them.

Owing to the high capability of gold NPs to be easily functionalized with numerous biomolecules, researchers have been developing novel approaches in cancer treatment, especially for PDT. In PDT, gold NPs’ utilization is based on the principle of surface functionalization with photosensitizers, which are then exposed to specific wavelengths of light to induce apoptosis. Gold NPs’ superior optical properties combined with their advantageous nature, including their high biocompatibility, inertness, resistance to chemical reactions, and low toxicity, contribute to the overall outcomes of these applications.

In conclusion, gold NPs have enormous potential for use in medicinal and diagnostic applications, and the worldwide market is predicted to achieve new heights. The transition of lab bench technology to real-world applications remains a gap, despite the abundance of research articles on the use of gold NPs in biomedical domains. Enhancements should concentrate on their transferability and thorough evaluation of the gold NP-based field in order to advance the field. This will minimize toxicity without sacrificing the effectiveness of treatments or diagnostics.

## Figures and Tables

**Table 3:** Recent PTT-based gold NP applications.

Application	Synthesis Method	Properties	Results	Reference
Selective destruction of cancer cells	Chemical synthesis	Average size of 204 nm. Nanostar morphology.	- Selective, site-specific destruction of HeLa, HEK-293, and SAOS-2 cell lines through laser irradiation.	[115]
Surface-enhanced Raman scattering (SERS) image-guided tumor PTT	Chemical synthesis	Average size of 35 nm. Spherical morphology. Peak at 521 nm (red-shifted to 548 nm with coating).	- Significant PTT activity with 808 nm laser irradiation in both *in vitro* and *in vivo*. - Enhanced therapeutic efficacy, achieving complete cure in mice by day 30.	[116]
Synergistic ionidamine release with PTT for anticancer activity	Chemical synthesis	Size of 5–30 nm. Spherical morphology.	- Enhanced cytotoxicity through the release of lonidamine and 808 nm laser irradiation. - Nanoparticle aggregation at tumor sites in mice with enhanced PTT effects.	[117]
PTT against drug-resistant cancer cells	Green synthesis by fabrication with histidine and carboxylated chitosan	Approximate size of 6.37 nm. SPR peak at 535 nm.	- 27.8% photothermal efficiency under 660 nm laser irradiation. - Significant reduction in cell viability through PTT, down to 10%, at concentrations above 0.5 mg/mL. - 90% reduction in tumor volume in mice treated with PTT.	[118]
PTT for cancer treatment with nucleic acid functionalization	Chemical synthesis	Approximate size of 13.7 nm. Spherical morphology. Absorption peak at 520 nm.	- PTT induction with 808 nm NIR laser irradiation in the presence of intracellular mRNAs. - Significant reduction in tumor growth in animal models.	[119]
PTT for cancer treatment with 2D self-assembled amphiphilic peptide modification	Chemical synthesis	Average size of 12.71 nm. Ellipsoid-like morphology. SPR peak at 520 nm (slight red-shift to 530 nm).	- Enhanced photothermal conversion efficiency from 19.79% to 27.42%. - High biocompatibility and low toxicity. - 79% death rate of MCF-7 cells under 808 nm laser irradiation.	[120]
Plasmonic PTT through synergistic drug release with PLGA NPs	Chemical synthesis	Spherical and nanostar morphology.	- Concentration and dose-dependent cytotoxicity against neuroblastoma cells. - Off/on-based triggered drug release for targeted delivery. - Enhanced cytotoxicity with 808 nm NIR laser irradiation. - Significant induction of ROS.	[121]
PTT-mediated multi-wavelength photomagnetic imaging (PMI)	Chemical synthesis	Size of 10 nm. Nanorod morphology. Peak absorption at 850 nm.	- Potential new model to make precise determination of the concentration of gold NPs in tumors. - Determination of PTT parameters, such as illumination power, duration, and wavelength, can be possible with PMI.	[122]
Combination of PTT and radiotherapy for breast cancer treatment	Green synthesis by using dopamine (DA)-conjugated alginate as a reducing and stabilizing agent	Mean size of 8.7 ± 1.3 nm. Spherical and monodisperse morphology. SPR peak at 540 nm.	- Highly biocompatible. - Simultaneous treatment of PTT (NIR) and radiotherapy (X-ray) enhanced cell viability reduction up to 35%. - Lowest rate of colony formation was observed in combined therapy (~0.37). - Increased ROS levels. - Significant reduction in tumor growth and volumes *in vivo*.	[123]
Combined antibacterial activity in dental resin delivery with PTT	Purchased	Approximate size of 20 nm. Spherical norphology (shell). Peak absorbance at 660 nm.	- MIC of gold NPs against *S.mutants* was determined as 100 μg/mL. - Significant reduction in OD values of *S.mutants* with light irradiation.	[124]
PTT with methotrexate delivery through dual-targeted NPs for colorectal cancer	Chemical synthesis	Size of 51.33 ± 5.70 nm. Spherical morphology (hollow). SPR peaks at 690 nm and between 800 and 820 nm.	- Negligible cytotoxicity. - Stabile drug release profile. - Significant reduction in tumor growths in both PTT (most significant) and non-PTT mice groups. - Larger necrotic region in tumor tissue of PTT-treated mice group.	[125]

**Table 4:** Recent gold NP-based PDT applications.

Application	Synthesis Methods	Properties	Results	Reference
Photo-eradication of methicillin-resistant *Staphylococcus aureus* biofilm	Green synthesis using the cell-free filtrate obtained from *Trichoderma koningii*	Two size averagely 15 ± 3 nm and 20 ± 3 nm. Spherical morphology.	- Enhanced photodestruction efficiency against biofilms. - Increased ROS production. - Approximately 100% destruction of biofilms.	[147]
PDT-based anticancer therapy	Chemical synthesis	Size of 120 nm. Star-like morphology.	- Enhanced anticancer activity with light treatment. - Increased ROS synthesis under 660 nm light irradiation.	[148]
PDT against *Staphylococcus aureus*	Chemical synthesis	Size of length 53.2 nm ± 1.8 nm and width 23.6 nm ± 1.3 nm. Nanorod morphology. Transversal and longitudinal peaks at 520 nm and 660 nm.	- Significant bactericidal activity through 525 (superior) and 660 nm light irradiation (near 100% reduction). - Agglomeration of NPs on bacteria surface.	[149]
PDT for hypoxic tumor	Chemical synthesis	Mean size of 3 nm. Nanocluster morphology. Absorption peak at 385 nm.	- Strong photosensitizing property. - Modified NPs selectively target cancer cells. - Significant cytotoxicity (decreased up to 40%) through ROS generation with 532 nm light irradiation.	[150]
SERS imaging integrated PTT/PDT	Chemical synthesis	Size of 40 nm and 17 nm in width. Nanorod morphology.	- 52.38% photothermal conversion efficiency. - Significant ROS generation under 808 nm laser irradiation. - Successful targeting and imaging of 4T1 cells both *in vitro* and *in vivo*. - Combined therapy reduced cell viability to less than 15% and demonstrated 86.2% apoptosis rate.	[151]
PDT against resistant bacteria	Chemical synthesis	Average size of 11.38 ± 4.38 nm. Spherical morphology. (Properties of bismuth–gold NP hybrid.)	- Significant bacterial reduction by PDT up to 46.57% (almost 2× higher than non-PDT treatment).	[152]
Combined therapy with PTT against breast cancer	Chemical synthesis	Size between 30 and 40 nm. Spherical morphology. SPR peak at 530 nm.	- Reduced cytotoxicity of MB by conjugation into NPs. - Significant cytotoxicity levels (to 10%) with combined therapy. - Strong cytotoxicity, including at low concentrations.	[153]
PDT-based anticancer activity through nanocomplex against melanoma	Chemical synthesis	Size of 13.58 nm. Spherical morphology. Absorption peak at 535 nm.	- Reduction in cell survival rate down to less than 20%. - Increased levels of lactate dehydrogenase (LDH) and caspase-3 and decreased levels of ATP and mitochondrial membrane potential.	[154]

**Table 5:** Recently developed gold NP-based LSPR and SERS sensors.

Application	Synthesis Methods	Properties	Results	Reference
Morphine quantification	Chemical synthesis	Approximately 4.13 nm sized particles. LSPR peaks at 532 nm. Negative surface charge.	- The linear range for detecting increased morphine concentration was determined to be between 0.01 and 1.0 µg/mL. - The limit of detection was determined to be 0.006 µg/mL. - Recovery range between 96.4 and 101.6% in real samples. - High specificity.	[174]
Development of highly sensitive label-free optical biosensor	Chemical synthesis	Average size of 10.1 ± 1.7 nm. Absorbance peak at 524 nm.	- Enhancement in performance with the involvement of thin glass substrates (1 mm). - The limit of detection for streptavidin was determined to be 3.2 × 10^−10^ M. - Limit of detection for dinitrophenyl antibodies determined as 5.8 × 10^−11^ M.	[175]
Detection of interleukin-6	Chemical synthesis	Size of 32.8 nm. Spherical morphology (shell). Absorbance peak at 779 nm.	- Colorimetric detection of interleukin-6 with a limit of 5 ng/mL. - Photothermal quantitative detection of interleukin-6 with a limit of detection of 0.3 ng/mL (20 times lower than naked eye detection). - Rapid and specific detection.	[176]
**SERS**				
Detection of serum dopamine	Chemical synthesis	Approximately 25 nm size. Spherical morphology (nanoshell). (Size considered by the increased nm after coating.)	- Successful dopamine detection but non-specific in a label-free SERS system. - A direct and sensitive detection of dopamine was observed under the Azo reaction. - Significant linear range 10^−3^–10^−12^ mol/L and low limit of detection 10^−12^ mol/L in serum sample.	[177]
Detection of biothiols	Chemical synthesis	Approximate size of 25 ± 2.3 nm (nanocomposite). Spherical morphology. Extinction peak at 530 nm (red-shifted peak at 545 nm).	- Enhanced detection of biothiols with SERS-based dye-conjugated gold NPs. - The limit of detection ranges between 10^−12^ and 10^−15^. - Enhanced cellular imaging through discrimination of cancer cells based on biothiol concentration.	[178]
Biosensor development through freeze-driven synthesis	Chemical synthesis	Predominant sizes of 20, 40, and 80 nm. Absorption peak at 520 nm (red-shifted to ~650 nm).	- The successful development of DNA hairpin-conjugated gold NPs enabled dual-mode detection using novel methods.	[179]
**Others**				
Visualization of tissue-specific distribution patterns of functional metabolites	Chemical synthesis	Approximately 27 nm size. Spherical morphology. 355 nm UV–VIS absorption. (Synthesis based on cited references in the paper.)	- Wide-ranging detection of pesticides. - Visualization of primary and secondary metabolites and mechanical damages between healthy and infected citrus leaves.	[180]
Detection of miRNA levels in raw milk samples	Chemical synthesis	Average size of 16 ± 1 nm. Spherical morphology.	- Sensitive and rapid miRNA detection from four different milk samples through color change.	[181]
Detection of sesame DNA in food	Chemical synthesis	Average size of 13.6 ± 1.6 and 15.2 ± 1.2 nm (15 nm used). Spherical morphology. Maximum absorbance ~527 nm (541 nm in non-sesame samples).	- High-specific, significant detection of sesame DNA in various food samples. - Easy determination by clear color changes.	[182]
Detection of hepatitis virus	Purchased	20 nm in size. Spherical morphology. Maximum absorbance at 520 nm (red-shifted to 550 nm).	- Colorimetric response of gold NP-DNA Walkers in the presence of hepatitis A virus target sequences. - Specific detection of the target sequence. - Approximate limit of detection by 200 copies/mL.	[183]
Detection of *Candida albicans*	Chemical synthesis	40 nm in size.	- Colorimetric detection of *Candida albicans* β-1,3-D-glucans aptamers. - Significant stability and non-aggregative behavior of gold NPs.	[184]

**Table 6:** Recent biological applications of gold NPs.

Application	Synthesis Methods	Properties	Results	Reference
**Antibacterial**				
Metabolomic and docking study of gold NP’s antimicrobial activity	Green synthesis using *Arthrospira platensis* extract	Mean size of 134.8 nm. Rod-shaped morphology.	- Antibacterial activity against *Streptococcus pneumoniae* was shown with 12 μg/mL MIC value. - The molecular docking study showed the docking score of *A. platensis*-derived compound by −6.84 kcal/mol against many residues.	[216]
Antibacterial activity against bovine locomotion disorders	Commercially purchased, synthesized with physical methods	5–40 nm size range. Spherical morphology.	- 6.25 mg/L concentration of gold NP treatment reduced bacterial cell viability between 40 and 50%. - Higher concentrations up to 50 mg/L increased the reduction to approximately 90%.	[217]
Antibacterial activity against both Gram-positive and Gram-negative bacteria	Green synthesis from *Lannea discolor*	Size between 30 and 97 nm. Flower-shaped. SPR peak at 316 nm.	- Antibacterial activity against *E. coli*, *S. aureus*, *K. pneumoniae*, *B. subtilis*, *P. aeruginosa*, with MIC values down to 7.81 µg/mL for *B. subtilis*, *K. pneumoniae*, *P. aeruginosa.*	[218]
Evaluation of antibacterial activity and colorimetric sensing	Green synthesis from *Equisetum diffusum* leaf extract	Average size of 56.5 ± 1.2 nm. Nanocube structure. LSPR peak at 539 nm.	- Significant antibacterial activity against *Listeria monocytogenes* and *Cronobacter sakazakii* with inhibition zones of 24 and 14 mm, respectively. - Detection of Pb2+ ions and photodegradation of methylene blue by 97% efficiency.	[219]
Evaluation of antibacterial activity and colorimetric sensing	Green synthesis from leaves extract *Fagonia arabica*	Size ranging from 20 to 60 nm. Spherical morphology. SPR peak at 535 nm.	- Significant antibacterial activity with an inhibition zone ranging from 14 to 18 mm. - Detection of Cd2+ ions with a limit of detection of 0.03 nM.	[220]
Antibacterial activity against *Salmonella typhimurium (S. typhimurium)*, one of the most important food pathogens	Green synthesis from *Jatropha curcas*	Average size of 17 nm. Predominantly spherical. SPR peak at 526 nm.	- Efficient bactericidal activity, in comparison to the plant extract alone, was observed against *S. typhimurium*. - Reduction in bacterial growth was observed at 18 μg/mL of gold NPs.	[221]
**Antifungal**				
Determination of antifungal activity	Green synthesis from aqueous extract of *Ricinus cummunis* leaves	Size between 15 and 20 nm. Predominantly spherical, and some triangular morphology. SPR peak at 550 nm.	- Against *Aspergillus fumigatus* and *Candida albicans* IC50 values of gold NPs were determined as 88.90 and 58.31 μg/mL, respectively. - Antibacterial and dye degeneration studies were also conducted.	[222]
Determination of antifungal activity	Green synthesis from *Callistemon viminalis* extracts	100 nm size. Spherical morphology. Absorption peak at 525 nm.	- The antifungal activity against multiple types of fungal strains with inhibition zones ranging from 8.5 to 10.5 mm. - Antibacterial activity was also evaluated.	[223]
Antifungal activity against multidrug-resistant fungus	Tricyclic microwave-assisted chemical synthesis	Size range of 9–55 nm. Spherical morphology. Absorption peak at 506 nm. (From three variants.)	- Zone of inhibition determined between 1.2 and 1.7 cm. - MIC values in the ranges 21–52, 24–46, and 44–102 µg/mL, depending on the gold NP formulation.	[224]
Determination of antifungal activity of functionalized gold NPs	Chemical synthesis	Near size of 7 nm. Spherical morphology. SPR peak at 515 nm.	- Conjugation of vancomycin onto gold NPs reduced the MIC value from 37.5 to 4.68 µg/mL and 75.0 to 1.5 µg/mL (difference from fungal strains). - Germination inhibition of fungal strains by 93% and 35%. - Increased ROS levels.	[225]
**Antiviral**				
Antiviral activity against human adenovirus serotype 5 (HAdV-5)	Green synthesis using fodder yeast	Size range of 10–23 nm. Irregular and spherical. SPR peak at 540 nm.	- Virucidal activity against HAdV-5 was observed with a reduction of −2.25 log10 50% tissue culture infection dose (TCID50).	[226]
Antiviral activity against herpes simplex virus-2 (HSV-2) by the use of gold NPs coated with poly(styrene sulfonate	Brust–Schriffin method	Size - Spherical morphology.	- Antiviral activity against HSV-2 was observed with an IC50 of 89.91 ng/mL.	[227]
Antiviral activity against white spot syndrome virus (WSSV)	Green synthesis from *Brevibacterium casei* (SOSIST-06)	Size ranging from 9.5 to 52.3 nm. Spherical and triangular morphologies.	- Dose-dependent antiviral activity was demonstrated against WSSV. - At a concentration of 320 μg/mL, NPs reduced the expression of viral gene VP-28.	[228]
**Wound Healing**				
Diabetes-induced wound healing activity with hydrogels	Chemical synthesis	Size of 14.15 ± 1.02 nm. Spherical morphology. Absorbance peak at 520 nm.	- Significant antibacterial activity against wound infections with >15mm inhibition zone and colony reduction near 0%. - Significant reduction in wound area by day 6 compared to the control group, ranging from 80.5 to 50.7% and 92.8%, respectively (*in vivo*). - Enhanced wound healing through immunoregulation and induction of angiogenesis. (Results demonstrate difference based on the loaded gold NP ratio, from 5% to 50%.)	[229]
Determination of wound healing potential of collagen-I-coated gold NPs	Chemical synthesis	Average size of ~19 ± 0.2 nm. Spherical morphology. Absorption peak at 524 nm.	- Coated gold NPs induced the proliferation of human skin fibroblast (HSF) cells, ranging from 135.21 ± 6.69% to 154.74 ± 9.72%. - Uncoated particles did not induce visible toxicity or alterations in HSF proliferation. - Wound closure rates of ~57.16 ± 1.31% and ~67.67 ± 1.67% for uncoated and coated particles, respectively, while the control group showed only 22.12 ± 1.22% wound closure at 24 h. - After 48 h, the wound closure rates increased to 91.01 ± 2.71%, 95.19 ± 1.67%, and 59.96 ± 2.66% for coated particles, uncoated particles, and control group, respectively.	[230]
Determination of wound healing activity	Green synthesis using *Bulbine frutescens* (L.) Wild	Various sizes between 51.82 ± 33.76 nm and 289.3 ± 88.68. Round, hexagonal, and triangular morphologies. Absorption peak at 550 nm. (Considered differences among 4 types of extracts.)	- Up to 31.40 ± 0.88% wound closure ratio compared to control group 9.63 ± 0.22%. - Increased cell viability to 116.40 ± 9.35%.	[231]
**Anti-inflammatory**				
Therapeutic effects by gold NPs on asthma treatment	Green synthesis from *Descurainia sophia* extract	Size range of 10–38 nm. Spherical morphology. Absorption peak at 537 nm.	- Gold NPs showed anti-inflammatory effects by reducing inflammatory markers IgE, PLA2, and total protein levels. - Following treatment with gold NPs, improved lung pathology was observed in sensitized rats.	[232]
Determination of anti-inflammatory activity with ginsenoside compound K (CK) loading	Green synthesis using probiotic bacteria, *Bifidobacterium animalis* subsp. *lactis.*	Size range of 10–25 nm. Spherical morphology.	- Inhibition of ROS activation, reduction in the expression of iNOS, COX-2, and pro-inflammatory cytokines IL-1β, IL-6, and TNF-α were observed in RAW 264.7 cells. - Reduction in inflammation was observed in kidney, lung, and liver tissues of mice, without toxicity, following administration of gold NPs.	[233]
**Antioxidant**				
Determination of antioxidant activity	Green synthesis from *Allium cepa L.* peel aqueous extract	Size ranging between 6.08 and 54.20 nm. Spherical morphology. SPR peak at 561.11 nm.	- The antioxidant activity was evaluated with 4 different assays, with IC50 values ranging between 165.3 ± 1.93 and 193.7 ± 0.36 µg/mL. - Antipathogenic and enzyme inhibition activities were also observed.	[234]
Antioxidant activity with carrying extran-graft-polyacrylamide polymer	Chemical synthesis	Size of 5.5 ± 2.0 nm. Spherical morphology.	- At the highest concentration (50 mg/L), 64.66% radical scavenging activity was observed. - Antimicrobial and antibiofilm activity were also demonstrated.	[235]
**Antidiabetic**				
Determination of therapeutic effects of nature-friendly synthesized gold NPs	Green synthesis using *Nepeta bodeana* Bunge leaf extract	Size range of 20–30 nm. Spherical morphology. SPR peak at 547 nm.	- Antidiabetic activity was demonstrated with 52% inhibition of α-amylase and 62% glucose uptake activity in yeast cells, at 300 µg/mL of gold NPs.	[236]
Demonstration of antidiabetic activity of gold NPs	Green synthesis using seaweed extracts (*Ulva linza*, *Ulva fasciata*, *Ulva intestinalis*, *Petalonia fascia*, and *Corallina officinalis*)	Average diameter of 9.02 ±1.7 nm. Spherical morphology. SPR peak at 540 nm.	- Gold NPs achieved maximum α-glucosidase inhibition of 90.6%, at a concentration of 100 mg/mL, with IC50 value of 0.078 ± 0.003 mg/mL. - In the same tested concentrations, NPs exhibited maximum inhibition of 87.4% against α-amylase, with IC50 of 0.312 ± 0.014 mg/mL.	[237]
